# Supplementary material for: Zishen Yutai Pill increased live births in advanced maternal age women: a randomized clinical trial
Source: Nat Commun. 2025 Dec 24;17:980. doi: 10.1038/s41467-025-67714-4 (PMC12848059; doi:10.1038/s41467-025-67714-4)
Supplement: Supplementary file 1 — Supplementary Information [file 41467_2025_67714_MOESM1_ESM.pdf]

Zishen Yutai Pill increased live births in advanced maternal age women: A randomized clinical trial

Supplementary Information

Table of Content

CONTENT		PAGE
**Supplementary Tables**		
	**Supplementary Table 1.** Predefined subgroup analysis stratified by age	**1-2**
	**Supplementary Table 2.** Pregnancy outcomes in patient with embryo transfer	**3**
	**Supplementary Table 3.** Combined analysis of two randomized clinical trials in intention to treat population	**4**
	**Supplementary Table 4.** Combined analysis of two randomized clinical trial study in per protocol population	**5**
	**Supplementary Table 5.** Maternal, fetal, and neonatal adverse events	**6-7**
	**Supplementary Table 6.** Descriptive analysis of maternal adverse events during treatment period at the preferred term (PT) level, excluding pregnancy, puerperium and perinatal conditions	**8-11**
	**Supplementary Table 7.** Characteristics of the participants at baseline	**12-16**
	**Supplementary Table 8.** Outcomes of controlled ovarian hyperstimulation and characteristics of embryo transfer	**17-19**
	**Supplementary Table 9.** Post hoc analyses using Cochran-Mantel-Haenszel method for center effects on live birth among intention-to-treat population	**20**
	**Supplementary Table 10.** Post hoc analyses using logistic regression among intention-to-treat population	**21**
	**Supplementary Table 11.** Post hoc analyses using logistic regression among intention-to-treat population, adjusted for age and center	**22-23**
**Supplementary Note 1**	**Study Protocol**	**24-61**
**Supplementary Note 2**	**Protocol revision history and major changes**	**62-66**
**Supplementary Note 3**	**Informed Consent and translation**	**67-81**
**Supplementary Note 4**	**List of participating sites, ethics committee and approval number**	**82-84**
**Supplementary Note 5**	**Study Drug**	**85-93**
**Supplementary Note 6**	**Funding information**	**94-100**

Supplementary Table 1. Predefined subgroup analysis stratified by age

Outcome	ZYP group	Placebo group	Relative Ratio (95% CI)	Absolute Difference between Groups (95% CI)	P
**Age 35-37**	**n=425**	**n=426**			
Live birth, n (%)	123 (28.9)	90 (21.1)	1.37 (1.08~1.74)	7.8 (2.0~13.6)	0.009
Biochemical pregnancy, n (%)	152 (35.8)	125 (29.3)	1.22 (1.00~1.48)	6.2 (0.1~12.7)	0.046
Implantation rate, no./total no. (%)	171/414 (41.3)	126/384 (32.8)	1.26 (1.05~1.51)	8.5 (1.8~15.1)	0.013
Clinical pregnancy, n (%)	145 (34.1)	111 (26.1)	1.31 (1.06~1.61)	8.1 (1.9~14.1)	0.010
Pregnancy loss, no./total no. (%)					
Among biochemical pregnancy	28/152 (18.4)	31/125 (24.8)	0.74 (0.47~1.17)	-6.4 (-16.2~3.3)	0.197
Among clinical pregnancy	22/145 (15.2)	21/111 (18.9)	0.80 (0.47~1.38)	-3.8 (-13.4~5.4)	0.427
First trimester	19/145 (13.1)	19/111 (17.1)	0.77 (0.43~1.38)	-4.0 (-13.3~4.7)	0.371
Second trimester	3/145 (2.1)	2/111 (1.8)	1.15 (0.20~6.76)	0.3 (-4.5~4.3)	1.000
**Age 38-39**	**n=213**	**n=212**			
Live birth, n (%)	38 (17.8)	37 (17.5)	1.02 (0.68~1.54)	0.4 (-6.9~7.7)	0.917
Biochemical pregnancy, n (%)	63 (29.6)	55 (25.9)	1.14 (0.84~1.55)	3.6 (-4.9~12.1)	0.403
Implantation rate, no./total no. (%)	65/196 (33.2)	61/201 (30.3)	1.09 (0.82~1.46)	2.8 (-6.3~11.9)	0.547
Clinical pregnancy, n (%)	57 (26.8)	52 (24.5)	1.09 (0.80~1.51)	2.2 (-6.1~10.5)	0.598
Pregnancy loss, no./total no. (%)					
Among biochemical pregnancy	22/63 (34.9)	18/55 (32.7)	1.07 (0.64~1.77)	2.2 (-14.7~18.7)	0.802
Among clinical pregnancy	19/57 (33.3)	15/52 (28.8)	1.16 (0.66~2.03)	4.5 (-12.8~21.2)	0.614
First trimester	17/57 (29.8)	13/52 (25.0)	1.19 (0.64~2.21)	4.8 (-11.9~21.0)	0.573
Second trimester	2/57 (3.5)	2/52 (3.8)	0.91 (0.13~6.25)	-0.3 (-9.8~11.2)	1.000
**Age 40-42**	**n=96**	**n=95**			

Live birth, n (%)	10 (10.4)	12 (12.6)	0.83 (0.37~1.82)	-2.2 (-11.6~7.1)	0.632
Biochemical pregnancy, n (%)	17 (17.7)	15 (15.8)	1.12 (0.60~2.11)	1.9 (-8.8~12.6)	0.723
Implantation rate, no./total no. (%)	16/90 (17.8)	17/82 (20.7)	0.86 (0.46~1.58)	-3.0 (-14.9~8.9)	0.623
Clinical pregnancy, n (%)	14 (14.6)	14 (14.7)	0.99 (0.50~2.00)	-0.2 (-10.4~10.0)	0.976
Pregnancy loss, no./total no. (%)					
Among biochemical pregnancy	7/17 (41.2)	2/15 (13.3)	3.01 (0.75~12.65)	27.8 (-7.8~55.5)	0.122
Among clinical pregnancy	4/14 (28.6)	2/14 (14.3)	2.00 (0.43~9.21)	14.3 (-20.9~46.0)	0.648
First trimester	3/14 (21.4)	2/14 (14.3)	1.50 (0.29~7.65)	7.1 (-26.3~39.1)	1.000
Second trimester	1/14 (7.1)	0	-	7.1 (-20.5~35.8)	1.000

To estimate the possible live birth increase, the live birth outcome of AMA women aged 35-39 was also analyzed. In the ITT analysis, ZYP intervention led a significant increase in the live birth rate among participants aged 35-39 (25.2% [161/638] vs. 19.9% [127/638], absolute difference, 5.3%; 95%CI, 0.7%~9.9%; P = 0.023).

Pregnancy outcomes were summarized with frequencies, percentages and RR and absolute difference of proportion with 95% CI. Between group comparisons were performed with $\chi 2$ test or Fisher's exact test, as appropriate. For RR, Delta method was used to calculate 95% CI. For absolute difference in proportions, Newcombe method was employed to calculate 95% CI. Two-sided p-values are reported; no adjustment for multiple comparisons was applied as all subgroup analyses were exploratory. ZYP, Zishen Yutai Pill; RR, relative ratio; CI, confidence interval.

Supplementary Table 2. Pregnancy outcomes in patient with embryo transfer

Outcome	ZYP group (n=422)	Placebo group (n=412)	Relative Ratio (95% CI)	Absolute Difference between Groups (95% CI)	P
Live birth, n (%)	171 (40.5)	139 (33.7)	1.20 (1.01~1.44)	6.8 (0.2~13.3)	0.043
Biochemical pregnancy, n (%)	232 (55.0)	195 (47.3)	1.16 (1.02~1.33)	7.7 (0.9~14.3)	0.027
Implantation rate, no./total no. (%)	252/700 (36.0)	204/667 (30.6)	1.18 (1.01~1.37)	5.4 (0.4~10.4)	0.034
Clinical pregnancy, n (%)	216 (51.2)	177 (43.0)	1.19 (1.03~1.38)	8.2 (1.5~14.9)	0.017
Pregnancy loss, no./total no. (%)					
Among biochemical pregnancy	57/232 (24.6)	51/195 (26.2)	0.94 (0.68~1.30)	-1.6 (-9.9~6.6)	0.707
Among clinical pregnancy†	45/216 (20.8)	38/177 (21.5)	0.97 (0.66~1.42)	-0.6 (-8.9~7.4)	0.878
First trimester	39/216 (18.1)	34/177 (19.2)	0.94 (0.62~1.42)	-1.2 (-9.0~6.5)	0.770
Second trimester	6/216 (2.8)	4/177 (2.3)	1.23 (0.35~4.29)	0.5 (-3.2~4.0)	1.000

Pregnancy outcomes were summarized with frequencies, percentages and RR and absolute difference of proportion with 95% CI. Between group comparisons were performed with $\chi 2$ test or Fisher's exact test, as appropriate. For RR, Delta method was used to calculate 95% CI. For absolute difference in proportions, Newcombe method was employed to calculate 95% CI. Two-sided p-values are reported; no adjustment for multiple comparisons was applied as all subgroup analyses were exploratory. ZYP, Zishen Yutai Pill; RR, relative ratio; CI, confidence interval.

Supplementary Table 3. Combined analysis of two randomized clinical trials in intention to treat population

Outcome	ZYP group (n=940)	Placebo group (n=954)	Relative Ratio (95% CI)	Absolute Difference between Groups (95% CI)	P
Live birth, n (%)	225 (23.9)	183 (19.2)	1.25 (1.05~1.48)	4.8 (1.1~8.5)	0.012
Biochemical pregnancy, n (%)	314 (33.4)	258 (27.0)	1.24 (1.08~1.42)	6.4 (2.2~10.5)	0.003
Implantation rate, no./total no. (%)	341/993 (34.3)	269/957 (28.1)	1.22 (1.07~1.40)	6.2 (2.1~10.3)	0.003
Clinical pregnancy, n (%)	284 (30.2)	228 (23.9)	1.26 (1.09~1.47)	6.3 (2.3~10.3)	0.002
Pregnancy loss, no./total no. (%)					
Among biochemical pregnancy	78/314 (24.8)	66/258 (25.6)	0.97 (0.73~1.29)	-0.7 (-7.9~6.3)	0.839
Among clinical pregnancy†	59/284 (20.8)	45/228 (19.7)	1.05 (0.74~1.49)	1.0 (-6.1~7.9)	0.772
First trimester	48/284 (16.9)	38/228 (16.7)	1.01 (0.69~1.50)	0.2 (-6.4~6.7)	0.944
Second trimester	9/284 (3.2)	7/228 (3.1)	1.03 (0.39~2.73)	0.0 (-3.4~3.3)	0.949

†In ZYP group, two case of pregnancy loss occurred, but when pregnancy loss occurred was unknown.

Pregnancy outcomes were summarized with frequencies, percentages and RR and absolute difference of proportion with 95% CI. Between group comparisons were performed with $\chi 2$ test or Fisher's exact test, as appropriate. For RR, Delta method was used to calculate 95% CI. For absolute difference in proportions, Newcombe method was employed to calculate 95% CI. Two-sided p-values are reported; no adjustment for multiple comparisons was applied as all subgroup analyses were exploratory. ZYP, Zishen Yutai Pill; RR, relative ratio; CI, confidence interval.

Supplementary Table 4. Combined analysis of two randomized clinical trial study in per protocol population

Outcome	ZYP group (n=503)	Placebo group (n=502)	Relative Ratio (95% CI)	Absolute Difference between Groups (95% CI)	P
Live birth, n (%)	207 (41.2)	170 (33.9)	1.22 (1.04~1.43)	7.3 (1.3~13.2)	0.017
Biochemical pregnancy, n (%)	288 (57.3)	239 (47.6)	1.20 (1.07~1.35)	9.7 (3.5~15.7)	0.002
Implantation rate, no./total no. (%)	316/893 (35.4)	253/887 (28.5)	1.24 (1.08~1.42)	6.9 (2.5~11.2)	0.002
Clinical pregnancy, n (%)	262 (52.1)	213 (42.4)	1.23 (1.08~1.40)	9.7 (3.5~15.7)	0.002
Pregnancy loss, no./total no. (%)					
Among biochemical pregnancy	74/288 (25.7)	63/239 (26.4)	0.98 (0.73~1.30)	-0.7 (-8.2~6.8)	0.862
Among clinical pregnancy†	55/262 (21.0)	43/213 (20.2)	1.04 (0.73~1.48)	0.8 (-6.6~8.0)	0.829
First trimester	44/262 (16.8)	36/213 (16.9)	1.00 (0.92~1.09)	-0.1 (-7.0~6.6)	0.975
Second trimester	9/262 (3.4)	7/213 (3.3)	1.05 (0.40~2.76)	0.2 (-3.6~3.6)	0.929

†In ZYP group, two case of pregnancy loss occurred, but when pregnancy loss occurred was unknown.

Pregnancy outcomes were summarized with frequencies, percentages and RR and absolute difference of proportion with 95% CI. Between group comparisons were performed with $\chi 2$ test or Fisher's exact test, as appropriate. For RR, Delta method was used to calculate 95% CI. For absolute difference in proportions, Newcombe method was employed to calculate 95% CI. Two-sided p-values are reported; no adjustment for multiple comparisons was applied as all subgroup analyses were exploratory. ZYP, Zishen Yutai Pill; RR, relative ratio; CI, confidence interval.

Supplementary Table 5. Maternal, fetal, and neonatal adverse events

**Adverse events, no. (%)**	**ZYP group**		**Placebo group**		**Relative Ratio (95% CI)**	**Absolute Difference between Groups (95% CI)**	**P**
	Denominator	Value	Denominator	Value			
Moderate or severe ovarian hyperstimulation syndrome (OHSS)	734	4 (0.5)	733	6 (0.8)	0.67 (0.19~2.35)	-0.3 (-1.3~0.7)	0.524
Ectopic pregnancy among biochemical pregnancies	232	4 (1.7)	195	5 (2.6)	0.67 (0.18~2.47)	-0.8 (-4.3~2.2)	0.738
Miscarriage-related hospitalization among biochemical pregnancies	232	12 (5.2)	195	7 (3.6)	1.44 (0.58~3.59)	1.6 (-2.7~5.7)	0.429
Pregnancy complications	216	26 (12.0)	177	24 (13.6)	0.89 (0.53~1.49)	-1.5 (-8.4~5.1)	0.652
Gestational diabetes		19 (8.8)		15 (8.5)	1.04 (0.54~1.98)	0.3 (-5.6~5.9)	0.910
Gestational hypertension		5 (2.3)		2 (1.1)	2.05 (0.40~10.43)	1.2 (-2.0~4.3)	0.465
Others†		4 (1.9)		8 (4.5)	0.41 (0.13~1.34)	-2.7 (-7.0~0.9)	0.126
Preterm delivery among live birth	171	30 (17.5)	139	22 (15.8)	1.11 (0.67~1.83)	1.7 (-6.9~9.9)	0.687
Low birth weight among neonates	197	43 (21.8)	161	34 (21.1)	1.03 (0.69~1.54)	0.7 (-8.0~9.1)	0.871
Congenital anomalies among neonates ‡	197	1 (0.5)	161	2 (1.2)	0.41 (0.04~4.45)	-0.7 (-3.9~1.8)	0.590
Neonates entering NICU	197	5 (2.5)	161	3 (1.9)	1.36 (0.33~5.61)	0.7 (-3.1~4.2)	0.735

ZYP: Zishen Yutai Pill. NICU, neonate intensive care unit.

† Other pregnancy complications include 5 cases of premature rupture of membrane (1 in ZYP and 4 in placebo), 2 cases of intrahepatic cholestasis of pregnancy (1 in ZYP and 1 in placebo), 1 case of gestational hypothyroidism in ZYP group, 1case of placenta accreta in ZYP group, 1 case of postpartum hemorrhage in placebo group, 1 case of placental abruption in placebo group, 1 case of placental infection in placebo group. In addition, one patient had both gestational diabetes and gestational hypertension in ZYP group; one patient had both intrahepatic cholestasis and gestational hypertension in ZYP group; one patient had both postpartum hemorrhage and gestational hypertension in placebo group.

‡ Two cases of congenital anomalies were observed in placebo group, including 1 congenital cerebral cyst, 1 congenital anomaly of external genitalia. One neonate had cryptorchidism and congenital atrial septal defect in ZYP group.

Pregnancy outcomes were summarized with frequencies, percentages and RR and absolute difference of proportion with 95% CI. Between group comparisons were performed with $\chi 2$ test or Fisher's exact test, as appropriate. For RR, Delta method was used to calculate 95% CI. For absolute difference in proportions, Newcombe method was employed to calculate 95% CI. Two-sided p-values are reported; no adjustment for multiple comparisons was applied as all subgroup analyses were exploratory. ZYP, Zishen Yutai Pill; RR, relative ratio; CI, confidence interval.

Supplementary Table 6. Descriptive analysis of maternal adverse events during treatment period at the preferred term (PT) level, excluding pregnancy, puerperium and perinatal conditions

SOC	PT	ZYP group (n=734)	Placebo group (n=733)
Patients with any AEs (excluding pregnancy, puerperium and perinatal conditions)		46 (6.27)	80 (10.91)
Blood and lymphatic system disorders			
	Granulocytopenia	0 (0)	1 (0.14)
	Hypercoagulation	0 (0)	1 (0.14)
Cardiac disorders			
	Palpitations	0 (0)	2 (0.27)
Endocrine disorders			
	Hyperprolactinaemia	5 (0.68)	1 (0.14)
	Hyperthyroidism	0 (0)	2 (0.27)
Eye disorders			
	Lacrimation increased	0 (0)	1 (0.14)
Gastrointestinal disorders			
	Abdominal distension	3 (0.41)	1 (0.14)
	Abdominal pain	6 (0.82)	3 (0.41)
	Ascites	0 (0)	2 (0.27)
	Constipation	3 (0.41)	4 (0.55)
	Diarrhea	0 (0)	3 (0.41)
	Dysgeusia	0 (0)	1 (0.14)
	Epigastric pain	0 (0)	2 (0.27)
	Gastrointestinal disorder	0 (0)	1 (0.14)
	Hemorrhoids	0 (0)	1 (0.14)

	Nausea	1 (0.14)	1 (0.14)
	Noninfective gingivitis	0 (0)	1 (0.14)
	Toothache	0 (0)	1 (0.14)
	Vomiting	3 (0.41)	5 (0.68)
General disorders and administration site conditions			
	Chest discomfort	0 (0)	1 (0.14)
	Asthenia	0 (0)	1 (0.14)
	Malaise	0 (0)	4 (0.55)
	Pyrexia	0 (0)	1 (0.14)
	Ulcer	0 (0)	1 (0.14)
Hepatobiliary disorders			
	Hepatic function abnormal	2 (0.27)	1 (0.14)
Immune system disorders			
	Hypersensitivity	0 (0)	1 (0.14)
Infections and infestations			
	Conjunctivitis	0 (0)	1 (0.14)
	Endometritis	0 (0)	2 (0.27)
	Nasopharyngitis	1 (0.14)	2 (0.27)
	Pelvic inflammatory disease	0 (0)	1 (0.14)
	Pharyngitis	2 (0.27)	1 (0.14)
	Tonsillitis	0 (0)	1 (0.14)
	Upper respiratory tract infection	3 (0.41)	12 (1.64)
	Vaginal infection	1 (0.14)	3 (0.41)
Injury, poisoning and procedural complications			
	Limb injury	1 (0.14)	0 (0)
	Post procedural hemorrhage	0 (0)	1 (0.14)

Investigations			
	Fibrin D dimer increased	0 (0)	1 (0.14)
Metabolism and nutrition disorders			
	Decreased appetite	0 (0)	1 (0.14)
Musculoskeletal and connective tissue disorders			
	Back pain	2 (0.27)	0 (0)
	Rheumatoid arthritis	0 (0)	1 (0.14)
Nervous system disorders			
	Dizziness	2 (0.27)	0 (0)
	Headache	2 (0.27)	3 (0.41)
Psychiatric disorders			
	Insomnia	4 (0.54)	3 (0.41)
Renal and urinary disorders			
	Renal function abnormal	1 (0.14)	0 (0)
Reproductive system and breast disorders			
	Cervix disorder	1 (0.14)	0 (0)
	Hydrometra	5 (0.68)	3 (0.41)
	Hydrosalpinx	1 (0.14)	3 (0.41)
	Menstrual disorder	0 (0)	1 (0.14)
	Ovarian cyst	1 (0.14)	0 (0)
	Pelvic effusion	1 (0.14)	0 (0)
	Vaginal bleeding	4 (0.54)	6 (0.82)
Respiratory, thoracic and mediastinal disorders			
	Cough	0 (0)	2 (0.27)
	Laryngeal pain	0 (0)	3 (0.41)
Skin and subcutaneous tissue disorders			

	Dermatitis allergic	0 (0)	2 (0.27)
	Dermatitis	1 (0.14)	0 (0)
	Urticaria	1 (0.14)	0 (0)
Vascular disorders			
	Hypertension	0 (0)	1 (0.14)

Note: Data are presented as n (%). This table summarizes maternal adverse events (AEs) reported during the treatment period, excluding pregnancy-, puerperium-, and perinatal-related conditions. For pregnancy-related AEs, please refer to Supplementary Table 5. Serious adverse events (SAE) are also available in Supplementary Table 5, including moderate or severe ovarian hyperstimulation syndrome (OHSS), ectopic pregnancy and miscarriage-related hospitalization. Pregnancy complications, as well as neonatal and fetal adverse events, were not included as AE here because they occurred several months after the end of the treatment period. All AEs were recorded during the treatment period. Events such as pregnancy complications, neonatal, and fetal outcomes were instead collected later during live birth data collection and are therefore reported separately from treatment-period AEs. In addition, two cases of non pregnancy-related SAEs (both resulting in hospitalization) were observed, one case of pneumonia in the placebo group and one appendicectomy in the ZYP group.

PT: preferred term. SOC: system organ class.

Supplementary Table 7. Characteristics of the participants at baseline

Characteristics	ZYP group (N=734)	Placebo group (N=733)
Age, years		
Mean±SD	37.3±1.8	37.3±1.9
Min–Max	34.0–42.0	33.0–43.0
Median (IQR)	37.0 (36.0–39.0)	37.0 (36.0–39.0)
Weight, kilogram		
Mean±SD	57.8±7.8	57.4±7.5
Min–Max	39.0–84.0	38.0–87.0
Median (IQR)	58.0 (52.0–63.0)	57.0 (52.0–62.0)
Height, centimeter		
Mean±SD	159.4±5.4	159.1±5.3
Min–Max	134.0–176.0	140.0–173.5
Median (IQR)	160.0 (156.0–163.0)	159.0 (156.0–163.0)
BMI, kg/m2		
Mean±SD	22.7±2.6	22.7±2.6
Min–Max	16.2–30.5	15.4–34.0
Median (IQR)	22.7 (20.8–24.4)	22.6 (20.9–24.3)
Type of infertility		
Primary infertility	181 (24.9)	171 (23.6)
Secondary infertility	545 (75.1)	554 (76.4)
Duration of attempt to conceive, years		
Mean±SD	4.7±3.9	4.9±3.9
Min–Max	1.0–21.0	1.0–21.0

Median (IQR)	3.0 (2.0–6.5)	4.0 (2.0–7.0)
Concomitant infertility factors		
Pelvic factors and tubal factors		
Yes	519 (70.7)	542 (73.9)
No	215 (29.3)	191 (26.1)
Endometriosis		
Yes	22 (3.0)	13 (1.8)
No	712 (97.0)	720 (98.2)
Male factors		
Yes	331 (45.1)	345 (47.1)
No	403 (54.9)	388 (52.9)
Unexplained factors		
Yes	35 (4.8)	25 (3.4)
No	699 (95.2)	708 (96.6)
Ovulation disorder		
Yes	78 (10.6)	96 (13.1)
No	656 (89.4)	637 (86.9)
Scar uterus		
Yes	88 (12.0)	93 (12.7)
No	646 (88.0)	640 (87.3)
Diabetes		
Yes	32 (4.4)	35 (4.8)
No	702 (95.6)	698 (95.2)
Obstetric history		

Gravidity		
Mean±SD	1.7±1.5	1.8±1.6
Min–Max	0.0-10.0	0.0-10.0
Median (IQR)	2 (1, 3)	2 (1, 3)
Parity		
Mean±SD	0.7±0.7	0.6±0.7
Min–Max	0.0-3.0	0.0-3.0
Median (IQR)	1 (0, 1)	1 (0, 1)
Abortion history		
Mean±SD	0.8±1.1	0.9±1.2
Min–Max	0.0-8.0	0.0-8.0
Median (IQR)	0 (0, 1)	1 (0, 1)
Previous IVF cycles		
0	604 (83.4)	591 (81.7)
1	54 (7.5)	71 (9.8)
2	48 (6.6)	49 (6.8)
≥3	18 (2.5)	12 (1.7)
Previous miscarriage		
Yes	138 (18.8)	148 (20.2)
No	596 (81.2)	585 (79.8)
AFC, no.		
Mean±SD	12.7±7.6	12.1±8.0
Min–Max	0.0–70.0	0.0–120.0
Median (IQR)	11.0 (7.0–16.0)	11.0 (8.0–15.0)

Basal sex hormones		
AMH (ng/mL)		
Mean±SD	2.8±2.2	2.7±2.8
Min–Max	0.0–18.8	0.0–42.3
Median (IQR)	2.1 (1.3–3.7)	2.1 (1.2–3.4)
E2 (pmol/L)		
Mean±SD	235.2±330.6	247.0±434.8
Min–Max	0.2–5391.2	4.1–7373.0
Median (IQR)	160.7 (114.0–238.2)	159.6 (110.1–248.9)
FSH (IU/L)		
Mean±SD	7.4±3.5	7.3±3.6
Min–Max	0.7–24.9	0.5–35.3
Median (IQR)	6.8 (5.4–8.6)	6.8 (5.3–8.5)
LH (IU/L)		
Mean±SD	4.6±3.4	4.9±5.7
Min–Max	0.1–56.8	0.1–126.4
Median (IQR)	4.1 (2.9–5.3)	4.1 (3.0–5.5)
PRL (mIU/L)		
Mean±SD	479.4±1520.7	406.4±757.3
Min–Max	6.6–31757.6	0.0–9113.9
Median (IQR)	281.9 (207.8–384.7)	283.7 (198.8–387.9)
T (nmol/L)		
Mean±SD	11.5±47.9	8.2±29.7

Min–Max	0.0–691.6	0.0–270.6
Median (IQR)	0.9 (0.6–1.4)	0.9 (0.6–1.4)

Categorical variables were summarized with frequencies and percentages. Normally distributed variables were presented as means and standard deviation (SD), Non-normally distributed variables were presented as medians and interquartile ranges (IQR). ZYP: Zishen Yutai Pill; IQR, interquartile; SD: standard deviation; BMI: body mass index; AFC: antral follicular count; IVF: in vitro fertilization; AMH: anti-Müllerian hormone; E2: estradiol; FSH: follicular-stimulating hormone; LH: luteinizing hormone; PRL: prolactin; T: testosterone.

Supplementary Table 8. Outcomes of controlled ovarian hyperstimulation and characteristics of embryo transfer

**Characteristics**	**ZYP group (N=734)**	**Placebo group (N=733)**
Protocol		
Antagonist protocol	193 (27.1)	199 (28.2)
Long protocol	518 (72.9)	507 (71.8)
Days of ovarian stimulation, days		
Mean±SD	11.6±4.4	11.4±3.8
Min–Max	4.0–65.0	2.0–47.0
Median (IQR)	11.0 (9.0–13.0)	11.0 (9.0–13.0)
Gonadotropin dose, IU		
Mean±SD	1750.6±1648.6	1625.9±1246.0
Min–Max	37.5–27487.5	37.5–6750.0
Median (IQR)	1575.0 (656.3–2612.5)	1500.0 (515.6–2475.0)
Estradiol on hCG trigger day, pmol/L		
Mean±SD	10502.4±7185.7	10618.6±7140.6
Min–Max	4.8–79532.6	349.4–48943.1
Median (IQR)	8826.4 (5794.9–13990)	9446.6 (5450.0–14441)
Progesterone on hCG trigger day, μg/L		
Mean±SD	0.8±0.6	0.8±0.5
Min–Max	0.0–5.3	0.1–6.0
Median (IQR)	0.7 (0.5–1.1)	0.7 (0.5–1.0)
Luteinizing hormone on hCG trigger day, IU/L		
Mean±SD	2.2±1.9	2.3±3.7
Min–Max	0.1–20.2	0.1–57.1

Median (IQR)	1.7 (1.1–2.6)	1.6 (1.0–2.6)
Endometrial thickness on hCG trigger day, mm		
Mean±SD	11.3±2.5	11.2±2.6
Min–Max	0.0–22.0	3.4–19.6
Median (IQR)	11.0 (9.5–12.8)	11.0 (9.3–12.8)
Number of oocytes retrieved, no.		
Mean±SD	9.9±5.6	9.7±5.4
Min–Max	1.0–32.0	0.0–31.0
Median (IQR)	9.0(5.0–13.0)	9.0(6.0–13.0)
Number of cleavage, no.		
Mean±SD	7.4±4.7	7.1±4.5
Min–Max	0.0–28.0	0.0–27.0
Median (IQR)	7.0 (4.0–10.0)	6.0 (4.0–10.0)
Number of 2PN fertilization, no.		
Mean±SD	6.5±4.2	6.2±4.4
Min–Max	0.0–28.0	0.0–55.0
Median (IQR)	6.0(3.0–9.0)	6.0(3.0–9.0)
Number of available embryo, no.		
Mean±SD	4.0±2.8	3.7±2.6
Min–Max	0.0–22.0	0.0–18.0
Median (IQR)	4.0 (2.0–5.0)	3.0 (2.0–5.0)
Number of high-quality embryos, no.		
Mean±SD	2.9±3.1	2.6±2.9
Min–Max	0.0–17.0	0.0–20.0

Median (IQR)	2.0 (0.0–4.0)	2.0 (0.0–4.0)
Method of fertilization†		
IVF	324 (76.8)	318 (77.2)
ICSI	75 (17.8)	63 (15.3)
IVF+ICSI	23 (5.5)	31 (7.5)
Number of embryos transferred†		
1	148 (35.1)	158 (38.3)
2	270 (64.0)	253 (61.4)
3	4 (0.9)	1 (0.2)
The day of embryo transfer†		
D2	5 (1.2)	2 (0.5)
D3	319 (75.6)	320 (77.7)
D4	2 (0.5)	4 (1.0)
D5	96 (22.7)	86 (20.9)

Categorical variables were summarized with frequencies and percentages. Normally distributed variables were presented as means and standard deviation (SD), Non-normally distributed variables were presented as medians and interquartile ranges (IQR). ZYP: Zishen Yutai Pill; IQR, interquartile; SD: standard deviation; hCG: human chorionic gonadotropin. IVF: in vitro fertilization; ICSI: intracytoplasmic sperm injection (IVF/ICSI)

†Denominators were participants who underwent embryo transfer, 422 in ZYP group and 412 in placebo group.

Supplementary Table 9. Post hoc analyses using Cochran-Mantel-Haenszel method for center effects on live birth among intention-to-treat population

Effect estimate (Relative Ratio)	95% CI	Breslow-Day test	CMH P
1.31	1.02~1.69	0.997	0.035

RR: relative ratio; CI: confidence interval, CMH: Cochran-Mantel-Haenszel.

Supplementary Table 10. Post hoc analyses using logistic regression among intention-to-treat population

Factors	OR (95%CI)	P
Treatment [a]	1.31 (1.02~1.69)	0.035
Center [b]	-	0.838
Center 2	1.01 (0.55~1.86)	
Center 3	0.77 (0.45~1.33)	
Center 4	1.13 (0.64~1.98)	
Center 5	1.18 (0.53~2.61)	
Center 6	1.18 (0.65~2.15)	
Center 7	0.83 (0.41~1.65)	
Center 8	0.72 (0.38~1.35)	
Center 9	0.81 (0.42~1.54)	
Center 10	1.13 (0.68~1.88)	
Center 11	0.93 (0.50~1.71)	
Center 12	1.05 (0.55~2.00)	

OR: odds ratio; ZYP: Zishen Yutai pill.

[a] The logistic regression model was adjusted for factors including treatment and center.

[b] Center 1 (Sun Yat-Sen Memorial Hospital of Sun Yat-Sen University) was used as the reference category. Center 2: Women and Children's Hospital of Chongqing Medical University; Center 3: Tangdu Hospital, the Fourth Military Medical University; Center 4: The First Affiliated Hospital of Zhengzhou University; Center 5: The First Hospital of Lanzhou University; Center 6: Liuzhou Maternity and Child Healthcare Hospital; Center 7: West China Second University Hospital, Sichuan University; Center8: The Affiliated Suzhou Hospital of Nanjing Medical University; Center 9: Northwest Women's and Children's Hospital; Center 10: Reproductive and Genetic Hospital of CITIC-Xiangya; Center 11: The Third Affiliated Hospital of Zhengzhou University; Center 12: Hospital for Reproduction Medicine Affiliated to Shandong University.

Supplementary Table 11. Post hoc analyses using logistic regression among intention-to-treat population, adjusted for age and center

Factors	OR (95%CI)	P
**Sensitivity analysis 1[a]**		
Treatment	1.33 (1.03, 1.72)	0.030
Age	0.81 (0.75, 0.88)	<0.001
Center [b]	-	0.766
**Sensitivity analysis 2[b]**		
Treatment	1.33 (1.03, 1.72)	0.028
Age	0.81 (0.75, 0.88)	<0.001
Center		0.742
**Sensitivity analysis 3[c]**		
**MI 1**		
Treatment	1.34 (1.04, 1.73)	0.025
Age	0.81 (0.76, 0.88)	<0.001
Center		0.720
**MI 2**		
Treatment	1.31 (1.02, 1.7)	0.036
Age	0.82 (0.76, 0.88)	<0.001
Center		0.706
**MI 3**		
Treatment	1.35 (1.05, 1.74)	0.021
Age	0.81 (0.75, 0.87)	<0.001
Center		0.661
**MI 4**		
Treatment	1.33 (1.03, 1.72)	0.030
Age	0.81 (0.75, 0.88)	<0.001

Center		0.766
**MI 5**		
Treatment	1.34 (1.04, 1.73)	0.026
Age	0.81 (0.75, 0.88)	<0.001
Center		0.699

OR: odds ratio; CI: confidence interval; MI: multiple imputation

[a] Logistic regression models included treatment group, center, and age (the stratification variable).

Sensitivity analysis 1 used the most conservative assumption (all missing data were imputed as no live birth, consistent with the primary analysis). Odds ratios and P values are reported for treatment and age, with overall P values for center.

[b] Sensitivity analysis 2 made no imputation in live birth outcomes. Odds ratios and P values are reported for treatment and age, with overall P values for center.

[c] Sensitivity analysis 3 used multiple imputation (five imputations) to estimate missing live birth outcomes. As shown in **Figure 1**, a total of 6 participants, 3 in each group, underwent embryo transfer but had no available records for pregnancy test, clinical pregnancy confirmation and live birth outcomes. The imputation model included treatment group, study center, age, body mass index (BMI), type of infertility, duration of infertility, and antral follicle count (AFC) as predictors. A fixed random seed was used to ensure reproducibility. All five imputed datasets are reported separately. Odd ratios and P value of both treatment and age are reported. Overall P value for center are reported. P values are presented as exact values except for age, as these are exceedingly small.

Supplementary Note 1: Study Protocol

Protocol

The Application of Zishen Yutai Pill in Advanced Maternal Aged Women Undergoing IVF-ET

ClinicalTrials.gov ID-NCT03703700 (https://clinicaltrials.gov/study/NCT03703700)

**Version 3**	
Date written	May 24, 2019
Date approved	May 30, 2019

Note: The Steering Committee and the Data and Safety Monitoring Board have discussed and revised this protocol thoroughly, including two major revisions. The final version was written on May 24, 2019, and was approved by the IRB of Sun Yat-Sen Memorial Hospital of Sun-Yat Sen University on May 30, 2019. After its IRB approval, this protocol has been implemented and adhered to without amendment. The Statistical Analysis Plan is contained within the protocol.

CONTENTS

1. Committee composition **1**
1.1 Protocol committee 1
1.2 Steering committee 1
1.3 Data coordination committee 3
1.4 Third-party data statistical unit 3
1.5 Sponsor and contact information 3
1.6 Publication committee 3
2. Background **4**
3. Objectives **5**
4. Participants **5**
4.1 Inclusion criteria 5
4.2. Exclusion criteria 5
4.3 Drop-out 6
5. Study design **6**
5.1 General design 6
5.2 Randomization and blinding 7
5.3 Intervention of IVF/ICSI and study drug 7
5.4 Intervention of study drug 8
6. Study procedures and visits **11**
6.1 General visit setting 11
6.2 Screening visit 13
6.3 Gn initiation visit 13
6.4 ET visit 13
6.5 Biochemical pregnancy test visit 14
6.6 Clinical pregnancy test visit 14
6.7 Delivery visit 14
7. Physical examination **15**
8. Transvaginal ultrasound scan **15**
9. Laboratory tests **15**
10. Outcome measures **15**

11. Timeline and recruitment plan 20
12. Statistical analysis plan 20
12.1 General statistical consideration 20
12.2 Sample size estimation 20
12.3 Type of analysis 21
12.4 Interim analysis 21
13. Adverse event reporting 21
13.1 Risks and discomforts 21
13.2 Adverse event definitions 23
13.2.1 Definition of adverse event 23
13.2.2 Definition of serious adverse event 24
13.3 Recording of adverse events 25
13.4 Causality and severity assessment 25
13.4.1 Causality assessment 25
13.4.2 Severity assessment 26
13.5 Reporting of serious adverse events and unanticipated problems 27
14. Concomitant medication 27
15. Monitoring 28
15.1 Data and Safety Monitoring Board 28
15.2 Ethics 29
16. Data handling and record-keeping 29
16.1 Data entry and electronic case report form (eCRF) 29
16.2 Data quality control and query management 29
16.3 Data security 30
16.4 Audit 30
16.5 Medical Coding 30
17. Publication policy 31
18. Acknowledgment section 31
19. Protocol revision history 31
20. References 32

1. Committee composition

1.1 Protocol committee

Table 1. Protocol Committee

Name	Affiliation	Email
Dongzi Yang	Sun Yat-Sen Memorial Hospital of Sun Yat-Sen University	yangdz@mail.sysu.edu.cn
Yu Li	Sun Yat-Sen Memorial Hospital of Sun Yat-Sen University	liyuliyu0922@163.com
Heping Zhang	Yale University	heping.zhang@yale.edu
Jie Zhou	Guangzhou Evidence-Based Medicine Tech Co., Ltd	zhouj@gz-ebm.com

1.2 Steering committee

Table 2. Steering Committee

Name	Affiliation	Email
Chair		
Dongzi Yang	Sun Yat-Sen Memorial Hospital of Sun Yat-Sen University	yangdz@mail.sysu.edu.cn
Co-investigators		
Heping Zhang	Yale University	heping.zhang@yale.edu
Yu Li	Sun Yat-Sen Memorial Hospital of Sun Yat-Sen University	liyuliyu0922@163.com

Site-investigators		
Hong Ye	Women and Children's Hospital of Chongqing Medical University	yehong1210@163.com
Xiaohong Wang	Tangdu Hospital, the Fourth Military Medical University	wangxh919@fmmu.edu.cn
Linli Hu	The First Affiliated Hospital of Zhengzhou University	hulinli1999@163.com
Xiaolin Ma	The First Hospital of Lanzhou University	maxl2005@123.com
Li Fan	Liuzhou Maternity and Child Healthcare Hospital	3641917@qq.com
Wei Huang	West China Second University Hospital, Sichuan University	hdyynfm@163.com
Hong Li	The Affiliated Suzhou Hospital of Nanjing Medical University	hongliszivf@163.com
Haiyan Bai	Northwest Women’s and Children's Hospital	bhy1212@126.com
Fei Gong	Reproductive and Genetic Hospital of CITIC-Xiangya	lj_0305@126.com
Yichun Guan	The Third Affiliated Hospital of Zhengzhou University	lisamayguan@163.com
Ze Wang	Hospital for Reproduction Medicine Affiliated to Shandong University	wangze0806@163.com

1.3 Data coordination committee

Prof. Heping Zhang at Yale University will lead the Data Coordination Committee consisting of the personnel from Sun Yat-Sen Memorial Hospital of Sun Yat-Sen University, including registering the trial at the Chinese Clinical Trial Registry (http://www.chictr.org/cn/) and ClinicalTrials.gov (http://www.ClinicalTrials.gov). The daily operation of the Data Coordination Center is fully executed by the personnel at Sun Yat-Sen Memorial Hospital.

1.4 Third-party data statistical unit

Table 3. Third-party data statistical unit

Name	Affiliation	Email
Jie Zhou	Guangzhou Evidence-Based Medicine Tech Co., Ltd	zhouj@gz-ebm.com

1.5 Sponsor and contact information

Table 4. Sponsor and contact information

Name	Affiliation	Email
Na Ning	Guangzhou Baiyunshan Zhongyi Pharmaceutical Co. Ltd.	ningna0821@163.com

1.6 Publication committee

Members of the publication committee include Dongzi Yang, Yu Li.

2. Background

The deferment of childbirth has emerged as a significant concern in recent years. Over the past forty years, the prevalence of primiparity at age of 35 years or older increased by 9 times (1). Advanced maternal age (AMA), defined as pregnancy at 35 years or older, is commonly linked with reduced fecundity (2,3). AMA patients are more inclined to the use of assisted reproductive technology (ART), including in vitro fertilization (IVF) and intracytoplasmic sperm injection (ICSI) (4). Nonetheless, their pregnancy outcomes are typically less favorable compared to their younger counterparts, as aging adversely affects both oocyte quality and ovarian reserve (5).

Traditional Chinese Medicine (TCM) has been employed for over two millennia in the treatment of a myriad of conditions, including infertility (6). A representative formula for the treatment of infertility is the Zishen Yutai Pill (ZYP). ZYP is a patent Chinese medicine invented by Professor Yuankai Luo (7). Since its market debut in 1983, ZYP was renowned for its therapeutic effects on threatened miscarriage and recurrent miscarriage. ZYP is consisted of fifteen Chinese medicine, including Cuscutae Semen, Ginseng Radix et Rhizoma, Dipsaci Radix, Taxilli Herba, Eucommiae Cortex, Morindae Officinalis Radix, Cervi Cornu Degelatinatum, Codonopsis Radix, Atractylodis Macrocephalae Rhizoma, Asini Corii Colla, Lycii Fructus, Rehmanniae Radix Praeparata, Polygoni Multiflori Radix Praeparata, Artemisiae Argyi Folium, and Amomi Fructus (8).

In previous clinical practice, the administration of ZYP was observed to enhance the ovulation and pregnancy rate, while decrease the miscarriage rate among patients with polycystic ovary syndrome (PCOS) (9). Another clinical report had suggested that oral intake of ZYP around the luteal phase could significantly increase the embryo implantation rate (10). Our previous multicenter, placebo-controlled, randomized clinical trial had suggested that the intervention of ZYP among advanced maternal aged women could significantly increase the clinical pregnancy rate during ART.

However, this analysis constituted a post hoc subgroup analysis, lacking the statistical power to detect the between-group difference. Therefore, the design of the current

study aims to provide data regarding the efficacy and safety of ZYP in IVF/ICSI for the AMA patients.

3. Objectives

The present study aims to investigate the efficacy and safety of ZYP on liver birth rates among AMA infertile women during IVF/ICSI-ET. The primary objective is to determine the difference of live birth rate between the ZYP and placebo group. The secondary outcomes will include counts and rates of oocytes/embryos (oocytes retrieved, 2 pro-nuclei zygotes, cleavage zygotes, available embryos, high-quality embryos), pregnancy outcomes (rates of biochemical pregnancy, implantation, clinical pregnancy and miscarriage), incidences of maternal, fetal and neonatal complications, and neonate information (newborn birthweight and length, congenital malformation).

4. Participants

4.1 Inclusion criteria

Participants who meet all of the following criteria will be included in this study.

(1) Infertile women aged between 35 and 42 years old.

(2) Intend to undergo IVF-ET (GnRH-a long protocol or GnRH-ant protocol).

(3) BMI<28 kg/m^2.

(4) Bilateral ovaries exist.

(5) Patients who voluntarily sign the informed consent and agreed to be followed up as required by the study protocol.

4.2. Exclusion criteria

Participants who meet one of the following criteria will be excluded in this study.

(1) Recurrent implantation failure (previous three times or more IVF/ICSI-ET failure).

(2) Adenomyosis, the uterine cavity line constricted by uterine fibroids.
(3) Untreated bilateral hydrosalpinx.
(4) Endometrial diseases that have not been cured.
(5) Known diseases that are not suitable for undergoing ART or at the present not suitable for pregnancy;
(6) Recent therapy (within one month) for infertility with TCM.

4.3 Drop-out

Participants will dropout when:
(1) subjects who have adverse events that cannot be tolerated.
(2) Severe breach of the protocol.
(3) Subjects who exit due to personal or unpredictable reasons (detailed information need to be recorded).
(4) Subjects considered inappropriate to continue to participate in the study for other medical reasons.

5. Study design

5.1 General design

It will be a multicenter, prospective, randomized (1:1 treatment ratio) clinical trial comparing the live birth rates in AMA women assigned to these treatment arms after a fresh ET in 1466 infertile patients undergoing IVF or ICSI. Patients will be randomized into either of two groups, receiving ZYP or the placebo during IVF/ICSI. All of the participants will receive standardized controlled ovarian hyperstimulation (COH) protocols, i.e., GnRH agonist (GnRH-a) long protocol or GnRH antagonist (GnRH-ant) protocol), as appropriate, and then undergo embryo transfer (ET).

5.2 Randomization and blinding

The participants will be randomized 1:1 to receive double-blind single-dummy monotherapy with placebo or ZYP using the permuted block randomization method. The randomization will be stratified by the age (35-37, 38-39 and 40-42) by following the Human Fertilisation and Embryology Authority (11). The web-based Interactive Response Technology system is managed by independent statisticians, guiding drug dispensing.

Patients, investigators and clinical staff performing this trial will be blinded to treatment allocation. The package, appearance and odor are the same in the placebo and ZYP. The allocation and detailed information of randomization will be kept by the independent statisticians managing the Interactive Response Technology system. Blinding will be maintained until the completion of the analysis. Unblinding will be allowed in case of a medical emergency. The cause and time of unblinding will be recorded in detail and signed by the treating physicians.

When all live birth information is retrieved, the computer system will disclose the allocation.

5.3 Intervention of IVF/ICSI and study drug

5.3.1 Controlled Ovarian Hyperstimulation (COH)

Two COH protocols will be applied.

1) GnRH-a long protocol: All subjects in two groups will undergo COH after down-regulation with long-acting or short-acting GnRH-a in midluteal phase. Hormone levels, including follicle-stimulating hormone (FSH), luteinizing hormone (LH) level and estradiol (E_2) will be measured on the gonadotropin (Gn) initiation day. Follicular development (diameter and counts) and endometrial thickness will also be evaluated by means of transvaginal ultrasonography. COH will be initiated when the serum LH < 5 IU/L and E_2 < 50 ng/ml or endometrial thickness < 5 mm. The initiation dose and total Gn dose will be adjusted according to a combined consideration of age, weight, basal hormone levels, antral follicular count (AFC) and

previous cycle response.

2) GnRH-ant protocol: Initiation will be conducted on day 2 to day 4 of menstrual cycle. Hormone levels, including FSH, LH and E_2 levels will be measured on the Gn initiation day. Follicular development (diameter and counts) and endometrial thickness will also be evaluated by means of transvaginal ultrasonography. The initiation dose and total Gn dose will be adjusted according to a combined consideration of age, weight, basal hormone levels, AFC and previous cycle response.

5.3.2 Ovulation monitoring

The mean diameter of all follicles and endometrial thickness will be monitored by means of ultrasonography, along with testing of serum hormonal levels, including FSH, LH, E_2 and progesterone (P_4) on the day 5 to day 6 after Gn initiation.

5.3.3 Oocyte retrieval and embryo transfer

When the diameter and counts of follicles meet the criteria (two leading follicles≥18 mm; or ≥3 follicles ≥ 17 mm; or ≥4 follicles ≥ 16 mm), ovulation will be triggered by injecting human chorionic gonadotropin (HCG). Testing of serum FSH, LH, E_2 and P_4 levels will be conducted on HCG injection day. Oocyte collection will be performed 36 hours later. Luteal support will be started on oocyte retrieval day. Fertilization will be achieved by conventional IVF or ICSI with the husband's semen. On day 3 to day 5 after oocyte retrieval, one to three embryos will be transferred.

High-quality embryos is defined according to Istanbul consensus and Gardner criteria (12, 13).

Day 2	4 cells, cell fragments <10%, no multi-nucleus
Day 3	8 cells, cell fragments <10%, no multi-nucleus
Day 5	stage 4 blastocyst, grade A inner cell mass, trophectoderm at grade A

5.4 Intervention of study drug

Eligible subjects will be allocated to receive ZYP or the placebo 3 times daily, 5 g

each time on down-regulation day (GnRH-a long protocol) or day 19 to day 23 of previous menstrual cycle (GnRH-ant protocol). Study drug will be suspended during the 1st day to the 4th day of menstrual cycle. Continuation of the study intervention will be determined by the serum β-HCG pregnancy test carried out two weeks after embryo transfer. Intervention of study drug will be stopped if the pregnancy test is negative. For patients with positive results on the pregnancy test, the study medicine will be given until a confirmed clinical pregnancy (five weeks after ET).

5.4.1 Zishen Yutai Pill

The manufacture of ZYP complies with the relevant requirements of law of China's Drug Administration and Good Manufacturing Practice (GMP), with approval from the China National Medical Products Administration (Permit No.Z44020008).

Table 5. Herbal drugs used in Zishen Yutai Pill (ZYP) and the origins and medicinal parts

Herbal drugs	Origin of natural medicine
Cuscutae Semen	Ripe dried seed of *Cuscuta Chinensis* Lam.
Ginseng Radix et Rhizoma	Dried root and rhizome of *Panax ginseng* C. A. Mey.
Dipsaci Radix	Dried root of *Dipsacus asper* Wall. ex DC.
Taxilli Herba	Dried leafy stem and branch of *Taxillus chinensis* (DC.) Danser
Eucommiae Cortex	Dried bark of *Eucommia ulmoides* Oilv.
Morindae Officinalis Radix	Dried root of *Marinda officinalis* How
Cervi Cornu Degelatinatum	Residue after water extraction of ossified antler of *Cervus nippon* Temminck
Codonopsis Radix	Dried root of *Codonopsis pilosula* (Franch.) Nannf.
Atractylodis Macrocephalae Rhizoma	Dried rhizome of *Atractylodes macrocephala* Koidz.
Asini Corii Colla	Solid glue prepared by stewing and concentrating from the hide of *Equus asinus* L.
Lycii Fructus	Dried ripe fruit of *Lycium barbarum* L.
Rehmanniae Radix Praeparata	Steamed and dried root of *Rehmannia glutinosa* (Gaertn.) DC.
Polygoni Multiflori Radix Praeparata	Steamed and dried root of *Polygonum multiflorum* Thunb.
Artemisiae Argyi Folium	Dried leaf of *Artemisia argyi* Lévl. et Vant.
Amomi Fructus	Dried fruit of *Amomum villosum* Lour.

5.4.2 Composition and manufacturing process of the placebo

1) Composition: Pregelatinized starch, microcrystalline cellulose, black iron oxide, refined honey, dextrin.

2) Manufacturing process: Pregelatinized starch, microcrystalline cellulose, and black iron oxide are mixed, crushed, and sieved. The refined honey is added to the mixed powder to make the wet pill with the required size. After drying, it is coated with a mixture of black iron oxide and talc powder, 3% dextrin solution, 75% ethanol solution, and refined honey. Finally, eligible pills are polished using Chinese insect wax, selected, and packaged.

5.4.3 Adherence of study drug

Patients' adherence of study drug will be assessed. An actual dosage above 80% of required drug dosage will be considered consistent with protocol requirements.

In order to record and improve the adherence of study drug, a diary card will be used. The diary card includes information of participants, study drug dosage, date of drug administered. In addition, each participant will be assigned a clinical research coordinator to assure the adherence of study drug and carry out follow-up.

5.4.4 Package and label of study drug

Both the ZYP and the placebo will be provided in the same label and appearance (written in Chinese).

Drug No.:

The Application of Zishen Yutai Pill in Aged Women Undergoing IVF-ET

（Clinical Use Only） **Dosage of one week**

【Action and Use】 Tonify *Kidney* and *Spleen*, invigorates *Qi* that nourishes *Blood*, placate the fetus and strengthens the body. Used in pregnancy loss due to deficiency of *Kidney* and *Spleen*, debility of *Chong* and *Ren* (treatment and prevention of threatened miscarriage and spontaneous miscarriage)。

【Package】 5 g/package.

【Dosage and Administration】 Administered orally with honey water or dilute salt water. 5 g (1 package) each time, tid.

【Intervention period】 11 weeks.

【Storage Condition】 Please keep in seal and away from moister.

【Caution】 Please keep the remaining drug and the package until the end of clinical trial. Stored away from children.

Manufactured by Guangzhou Baiyunshan Zhongyi Pharmaceutical Co. Ltd.

Expired Date: ** Batch No.: **

6. Study procedures and visits

6.1 General visit setting

Briefly, five visit points are scheduled for this study. Visit 1 will be the screening visit. Visit 2 will be performed on the day of Gn initiation. Visit 3 will be carried out on the day of ET. Visit 4 will be carried out two weeks after ET to confirm biochemical pregnancy. Visit 5 will carried out five weeks after ET to confirm clinical pregnancy. Follow-up after delivery will be conducted through web or telephone contact to ascertain the live birth outcomes of participants (**Figure 1**).

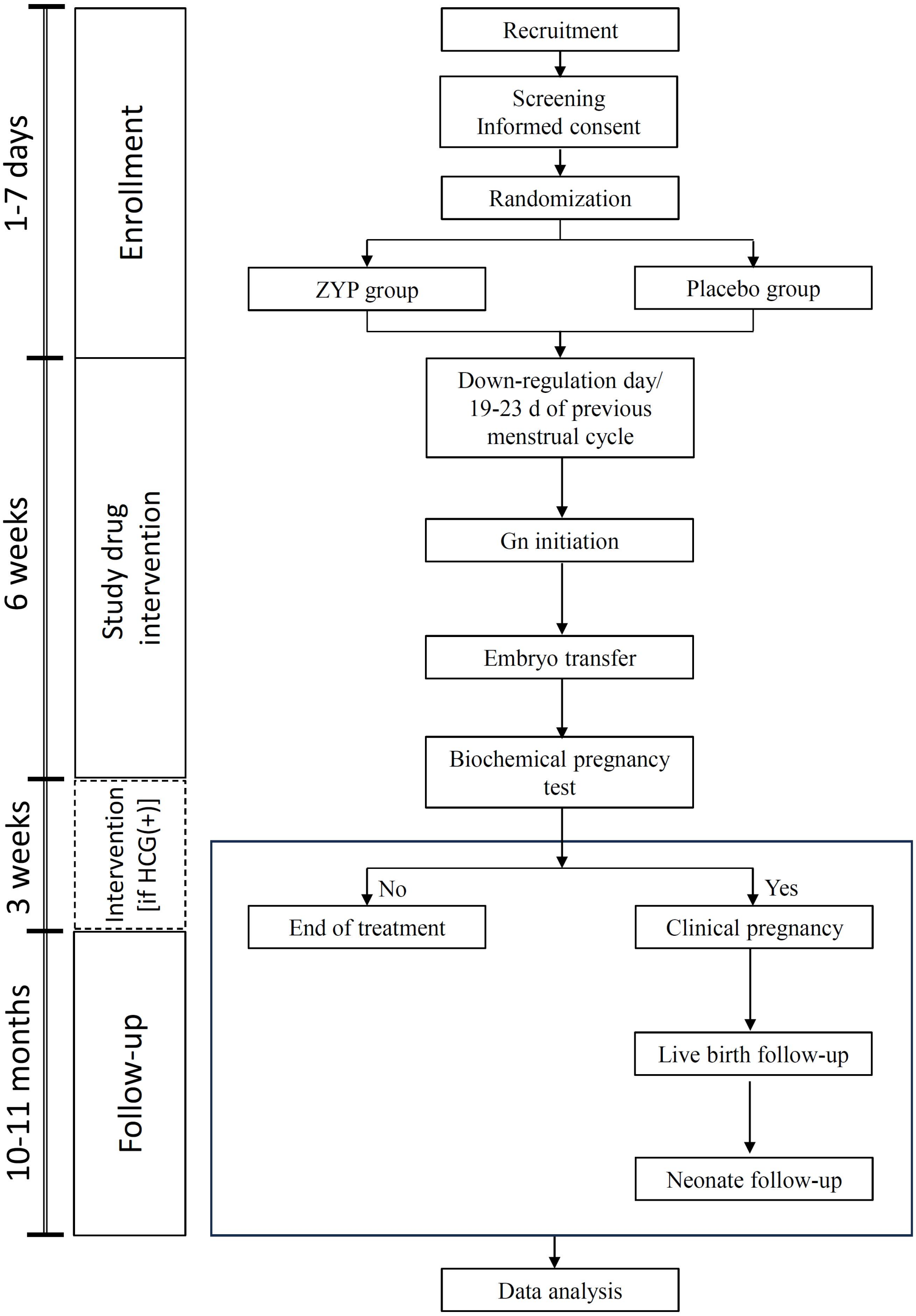

Figure 1. Study flowchart. Gn, gonadotropin; hCG, human chorionic gonadotropin; ZYP, Zishen Yutai Pill.

6.2 Screening visit

If the patient express interest toward the clinical trial, a screening visit will be performed. Patients will be informed about the study thoroughly. During the screening visit, the following procedures will be completed.

(1) Going through all the inclusion and exclusion criteria. Obtaining the signed informed consent form.

(2) Collecting baseline information, including age, medical record number, and birth date.

(3) Reviewing the medical history, including infertility history, childbearing history, gynecological surgery history, disease history and menstrual information.

(4) Carrying out a physical examination and gynecologic examination.

(5) Measuring the baseline sex hormones, including E_2, FSH, LH, prolactin (PRL), and testosterone (T).

(6) Reviewing the previous ET/FET cycle record.

(7) Dispensing information.

6.3 Gn initiation visit

During the Gn initiation visit, the following procedures will be completed.

(1) Adverse events and treatment (if necessary).

(2) Medication on Gn initiation, including type, name, initiation dose.

(3) Serum hormone levels, including E_2, FSH, LH, and P_4.

(4) Dispensing and return information.

6.4 ET visit

During the ET visit, the following procedures will be completed.

(1) Adverse events and treatment (if necessary).

(2) Summary of COH including type, name and total amount.

(3) B-mode ultrasound and basal serum hormonal level.

(4) Information about semen collection, embryo development, embryo transfer information.

(5) Information about the medicines used in luteal phase support, including name, dose, duration, and total amount.

(6) Dispensing and return information.

6.5 Biochemical pregnancy test visit

At 14 days after transplantation, a biochemical pregnancy test will be performed.

(1) Adverse events and treatment (if necessary).
(2) Serum level of β-HCG pregnancy test (β-HCG >50 IU/L is defined as biochemical pregnancy).
(3) Drug dispensing and return information will be recorded.

6.6 Clinical pregnancy test visit

If the pregnancy test is positive, transvaginal ultrasonography will be performed 3 weeks later to confirm clinical pregnancy.

(1) Adverse events and treatment (if necessary).
(2) Clinical pregnancy will be confirmed using B-mode ultrasound. Clinical pregnancy is defined as the presence of intrauterine gestation sac with fetal motion under transvaginal ultrasonography.
(3) Drug return information will be recorded.
(4) If a clinical pregnancy is confirmed, contact information and intention of delivery hospital will be checked.

6.7 Delivery visit

When the participant is preparing to deliver, the investigator will collect the delivery information and infant information according to the form designed for this visit. The delivery information mainly includes the delivery mode and pregnancy complications. The neonate information mainly includes gender, birth weight and length, birth

defects, admission to newborn intense care unit within 1 month and stillbirth.

7. Physical examination

A physical examination will be conducted on all participants by the corresponding investigator. The parameters include height, weight and they will be recorded to the nearest 0.1 cm, 0.1 kg. Height and weight will be measured without shoes, and patients will be dressed in light clothing.

8. Transvaginal ultrasound scan

An ultrasound scan with a transvaginal probe will be carried out. Uterine dimension, endometrial thickness and type, bilateral ovarian dimension, and follicle number will be measured through the ultrasound scan. The uterine size will be determined at the widest diameter. The investigator will determine endometrial types. Endometrial thickness is the largest anterior-posterior measurement of the endometrium in the sagittal plane. Ovarian dimension is measured by analysis of the largest plane of the ovary in two dimensions. In addition, antral follicle count (AFC) will be recorded.

9. Laboratory tests

Hormone and pregnancy tests will be performed at the local laboratories. In addition, blood work will be performed on screening visit, Gn initiation visit, hCG trigger day, and 2 weeks after ET. The blood sample (5 ml) will be collected with an anticoagulant tube and stored at -80°C for further analysis.

10. Outcome measures

The live birth rate is the primary outcome. The secondary outcomes will include counts and rates of oocytes/embryos (oocytes retrieved, 2 pro-nuclei zygotes, cleavage zygotes, available embryos, high-quality embryos), pregnancy outcomes (rates of biochemical pregnancy, implantation, clinical pregnancy and miscarriage),

incidences of maternal, fetal and neonatal complications, neonate information (newborn birthweight and length, congenital malformation) (14).

Live birth is defined as delivery of any viable infants after 28 weeks of gestation.

The number of oocytes retrieved will be defined as the total number of oocytes retrieved through ultrasound-guided transvaginal aspiration. The rate of oocytes retrieved will be defined as the number of retrieved oocytes divided by the number of follicles with a diameter ≥10mm on the HCG injection day. Matured oocytes will be defined as those with pronuclei or polar bodies observed on Day 1 after oocyte retrieval in IVF patients, or as the number of MII oocytes on the day of oocyte retrieval in ICSI patients. Cleavage oocytes will be defined as the number of fertilized oocytes that undergo cleavage on Day 2 after oocyte retrieval. The rate of cleavage will be defined as the number of cleaved embryos divided by the number of fertilized oocytes (2PN + 1PN + multiple PN). Available embryos will be defined as the sum of the number of embryos transferred and the number of embryos frozen. The rate of available embryos will be defined as the number of available embryos divided by the number of cleaved oocytes. High-quality embryo will be defined according to the day of embryo transfer, following the Istanbul consensus and Gardner criteria, Day 2: 4 cells, cell fragments <10% and no multi-nucleus; Day 3: 8 cells, cell fragments <10%, no multi-nucleus; Day 5: stage 4 blastocyst, grade A inner cell mass, grade A trophectoderm (12,13). The rate of high-quality embryos will be defined as the number of high-quality embryos divided by the number of available embryos.

Biochemical pregnancy will be defined as positive when β-HCG >50 IU/L. Implantation rate will be defined as the number of gestational sacs per the number of embryos transferred. Clinical pregnancy will be defined as the presence of intrauterine gestation sac with fetal motion under transvaginal ultrasonography. Miscarriage rate will be calculated among patients with biochemical pregnancies and patients with clinical pregnancies.

Incidences of maternal, fetal and neonatal adverse events were assessed, including moderate or severe OHSS, gestational diabetes mellitus (GDM), gestational

hypertension, postpartum hemorrhage, preterm delivery, congenital anomalies, puerperal infection, stillbirth, neonatal jaundice, neonatal infection, neonatal death, ectopic pregnancy, and low birth weight infant. The definitions of these safety indexes were shown in **Table 6**.

Table 6. Definitions of safety outcomes

Safety outcome	Definitions
Ovarian hyperstimulation syndrome (OHSS)	OHSS is defined according to the Golan criteria. Mild OHSS is diagnosed by the presence of abdominal distension and discomfort with or without nausea, vomiting, and/or diarrhea. Moderate OHSS is diagnosed when ultrasonographic ascites were present in addition to the above features. Severe OHSS is diagnosed when there is clinical evidence of ascites and/or hydrothorax or breathing difficulties with or without hemoconcentration, coagulation abnormalities, and diminished renal function.
Pregnancy loss	Pregnancy loss is defined as pregnancies that eventuate in spontaneous abortion or therapeutic abortion that occurred throughout pregnancy.
Gestational diabetes mellitus	GDM is defined as carbohydrate intolerance of variable severity with onset or first recognition during pregnancy as determined from the diagnosis in the obstetrical medical record.
Gestational hypertension	Gestational hypertension is defined as blood pressure ≥140/90 mmHg with negative proteinuria, which appeared during pregnancy and returned to normal 12 weeks after delivery. It may have upper abdominal discomfort or thrombocytopenia.
Postpartum hemorrhage	Postpartum hemorrhage is defined as the loss of 500 ml of blood or more after completion of the third stage of labor.
Preterm delivery	Delivery of a fetus at less than 37 and more than 28 weeks gestational age.
Congenital anomalies	Congenital anomalies are defined as structural or functional anomalies that occur during intrauterine life, including minor and major anomalies.
Puerperal infection	Any bacterial infection of the genital tract after delivery and during puerperium.

Stillbirth	The absence of signs of life at or after birth.
Neonatal jaundice	Neonatal jaundice is yellowing of the skin and other tissues of a newborn infant.
Neonatal infection	Neonatal infection is defined as a variety of infections in neonates caused by bacteria, fungi, viruses, etc., as determined from the diagnosis in the neonatal medical record.
Neonatal death	The death of a live-born neonate within 28 days after delivery.
Low birth weight infant	Neonatal birth weight ≤ 2500 g.
Ectopic pregnancy	Ectopic pregnancy is one in which the blastocyst implants at any site other than the endometrial lining of the uterus cavity.

11. Timeline and recruitment plan

The planned duration of recruitment will be 24 months with 12 centers. The enrollment target of our clinical trials is 1466 randomized participants. The number of participants taken in each center will be allocated according to the actual situation. The treatment period may need about 3 months and another 9 months to trace the pregnancy outcome. In short, a total of 36 months will be required to complete this trial, from initial recruitment to pregnancy outcome period.

12. Statistical analysis plan

12.1 General statistical consideration

For continuous variables, the Kolmogorov-Smirnov test will be applied to evaluate the distribution. Variables with normal distribution will be presented as means and standard deviation (SD), and intergroup comparisons will be performed using the 2-tailed, student's t test. Variables with non-normal distribution will be presented as medians and interquartile ranges (IQR), and intergroup comparisons will be performed using the Mann-Whitney U test. Categorical variables will be summarized with frequencies and percentages and compared using chi-square test or Fisher exact test, as appropriate. All statistical analysis will be conducted with the use of the statistical package SPSS, version 19.0 (SPSS Inc). A two-sided P value of <0.05 will be considered as significantly different.

12.2 Sample size estimation

In our previous clinical research, the live birth rate was 0.42 per embryo transfer in ZYP treated group and 0.33 per embryo transfer in placebo control group among AMA women. By assuming the same live birth rates, a sample size of 454 women in each group can provide a power of 80% at a significance level of 0.05. According to previous study, the dropout rate before ET was 15% and the cycle cancellation rate was 23% in AMA women. For this trial, a total rate of 38% (dropout and cancellation) is considered in the sample size calculation. Therefore, a total of 1466 participants

will be recruited in this study, 733 participants in each group.

12.3 Type of analysis

We will utilize an intent-to-treat approach to examine differences in the live birth rates for the first embryo transfer cycle in the two treatment arms in the primary analysis by the Pearson $\chi2$ test. Safety parameters and secondary efficacy parameters, such as pregnancy rate, miscarriage rate and other rates, will be analyzed using the Pearson $\chi2$ test or Fisher exact test as appropriate. Secondary efficacy parameters, including the counts and rates of oocytes/embryos, will be analyzed using the Mann-Whitney U test.

For the primary and secondary outcomes, we will also perform analysis in the per-protocol set (PPS), excluding those who have major protocol deviation(s), cancel cycle or do not complete the pre-set minimum exposure dosage of the assigned study drug (at least 80% compliance), from the ITT population. Subgroup analysis will be conducted as stratified by age (35-37, 38-39, 40-42).

Prior to unblinding, missing value in the primary outcome (live birth) and secondary outcomes (biochemical pregnancy, clinical pregnancy) in the ITT analysis will be imputed as not having an event.

12.4 Interim analysis

No interim analysis is planned.

13. Adverse event reporting

13.1 Risks and discomforts

Compared with the usual IVF patients, participating in this study will not increase additional risks. The possible risks and discomforts in common IVF technology are detailed in the informed consent, including in vitro fertilization ET, embryo freezing, and so on. The table below lists all procedures, including related risks and discomforts.

Table 7. Potential risks and discomfort of intervention during study process

Procedures and events	Risks and discomfort
Controlled ovarian hyperstimulation (COH)	Frequent subcutaneous injection, frequent venipuncture, frequent transvaginal ultrasound scan. Supra-physiologic E_2 may increase the risk of cancer ovary torsion or ovary rupture.
Ovarian hyperstimulation syndrome (OHSS)	Massive enlargement of the ovaries, ascites, bloating, nausea, and vomiting. Severe cases may have thoracic edema, breathing difficulties, oliguria, even anuria, and may require hospitalization, medication, or puncture drainage. A very severe case may suffer from thrombosis, damage to the liver or renal function, and even death.
Oocyte retrieval	Anesthesia accident, pelvic organ injury, intra-abdominal hemorrhage, puncture site hemorrhage, in serious case surgery or transfusion may be needed, infection.
ICSI	Microinjection may injure an oocyte, pass an unknown disease gene to the next generation.
Embryo transfer	Infection.
Embryo frozen and thaw	Embryotic development arrest. The survival rate of thawed embryos is 95%.
Standard venipuncture for blood work	Slight pain, ecchymosis at the site of puncture, infection, or bleeding at the site.
Transvaginal ultrasound	Abdominal or pelvic discomfort.
Ectopic pregnancy	May require medical or surgical treatment. In severe cases, pregnancy site rupture can result in intra-abdominal hemorrhage, even shock, or death if treatment is delayed.
Multiple pregnancies	May require embryo reduction, increase risk of pregnancy

	complication, fetus abnormalities, and preterm delivery.
Infertility treatment	Anxiety or emotional distress to various degrees.
Zishen Yutai Pills	It has been reported that some patients who took the Zishen Yutai pill suffered from nausea, dry mouth, and constipation that disappeared after drug withdrawal.

The participants are not expected to have all of these complications, and they will be allocated to a treatment group at random. The treatment may be less effective or have more complications than the other research treatment.

In this study, the GnRH-a long protocol and GnRH-ant protocol will be performed according to each patient's actual situation. The GnRH-ant protocol will be used to minimize the risk of OHSS. The initial dose will be determined according to the age, basal FSH level, basal AFC, weight, and previous situation promoting ovulation. The initiation dose and total Gn dose will be adjusted according to a combined consideration of age, weight, basal hormone levels, AFC and previous cycle response. In addition, in case of high ovarian response, the cycle will be canceled to avoid OHSS. If there are three or more fetuses, then a reduction will be performed to minimize the risks of multiple pregnancies. A responsible investigator or a resident doctor on 24 h call can be contacted at each site if any adverse event occurs during this study.

Every effort will be taken to avoid injury as a result of participation. If adverse events occur, active treatment will be provided. Furthermore, if a medical dispute is involved, it will be disposed of as a routine medical event.

13.2 Adverse event definitions

13.2.1 Definition of adverse event

Adverse event means any untoward or unfavorable medical occurrence associated with the subject's participation in the research, whether or not considered related to the study intervention.

Adverse events can be any of the following:

• Physical signs or symptoms, including medication side effects.

• Abnormal laboratory values.

• Changes in vital signs, physical exam findings, or test results.

• An increase in the frequency or intensity (worsening) of a condition or illness presents before study enrollment.

Note: In this trial, adverse events will not include:

• Pre-existing conditions or illnesses that do not worsen during the study period (record these in the medical history).

• Normal conditions associated with pregnancy.

13.2.2 Definition of serious adverse event

Serious adverse event: Any event temporally associated with the subject's participation in research that meets any of the following criteria:

• Death.

• Life-threatening (at immediate risk of death).

• Severely or permanently disabling.

• Requires in-patient hospitalization or prolongation of existing hospitalization.

• Pregnancy loss after 20 weeks gestation.

• Results in a congenital anomaly/birth defect.

• Or any event so deemed as serious by the PI at the site.

Note: A "severe" adverse event is not the same as a "serious adverse event" or SAE. Severity is based on the event's intensity, whereas seriousness is based upon the event outcome as it poses a threat to the patient's life or functioning.

13.2.3 Definition of Serious Unexpected Suspected Adverse Reaction (SUSAR)

SUSAR is defined as an serious adverse reaction that is both unexpected and meets the definition of an serious adverse reaction.

An adverse event is considered "unexpected" if its characteristic or severity exceed

current available product information, including the instruction of product or Investigator's Brochure.

13.3 Recording of adverse events

All adverse events will be observed during the clinical trial. The investigators will require the participants to reflect the change in patients' condition truthfully after using the drug and avoid suggestive questions. Adverse events and unexpected side effects (including symptoms, signs, and laboratory tests) will be observed while observing the curative effect. In order to determine whether adverse events are associated with the experimental drug, they will be recorded in the eCRF in detail, including the occurrence time, symptoms, signs, degrees, duration, laboratory examination indicators, treatment methods, procedures, results, follow-up time, and so on. Concomitant medication will be recorded in detail to analyze the correlation between adverse events and experimental drugs. In addition, the record will be signed and dated.

When adverse reactions occur, the investigator will take necessary measures, such as adjusting the dose, temporarily discontinuing the medication, and decide whether to terminate the trial or not. If a serious adverse event occurs, the unit undertaking the study must immediately take necessary treatment measures to ensure the subject's safety.

13.4 Causality and severity assessment

13.4.1 Causality assessment

According to the documented adverse events and abnormal test findings, the investigator will need to determine that if the abnormal test finding should be classified as an adverse event and if the adverse events are related to the study intervention or meet the criteria for a serious adverse event. The relationship between the experimental drug and adverse events are divided as "related", "probably related", "possibly related", "possibly unrelated", and "unrelated". AEs with the former three

kinds of relations are defined as adverse drug reactions (ADRs). The considerations of causality analysis include five aspects:

(1) There is a reasonable chronological relation between drug administration and the occurrence of suspected ADRs (occurrence after drug administration).

(2) Suspected ADRs are in accordance with known ADRs of the drug (literature compliance).

(3) Suspected ADRs cannot be explained by concomitant medication, previous medication, existing clinical conditions of patients, or the effects of other therapies (other explanation).

(4) After drug discontinuation or dose reduction, suspected ADRs disappear or relieve (disappearance after drug discontinuation).

(5) Suspected ADRs recur after re-exposure to the same drug (Recurrence after re-administration).

The investigators should assess the possible relation between AEs and the research drug or the concomitant medication.

Consideration	Occurrence after drug administration	Literature compliance	Other explanation	Disappearance after drug discontinuation	Recurrence after re-administration
Related	+	+	-	+	+
Probably related	+	+	-	+	?
Possibly related	+	+	±	±	?
Possibly unrelated	+	-	±	±	?
Unrelated	-	-	+	-	-

13.4.2 Severity assessment

The level of adverse event response will be evaluated and reported as follows:

Mild: The participant can tolerate the event, and it does not affect the treatment. It need not take special actions and is not harmful to the participant.

Moderate: The participant is intolerant and requires withdrawal or special treatment, which has a direct impact on their health.

Severe: It is a life-threatening, fatal, or disabling event and requires withdrawal or emergency treatment immediately.

13.5 Reporting of serious adverse events and unanticipated problems

Whether or not related to the study drugs, serious adverse events during the trial should be treated and reported to the primary investigator in the trial center promptly. Moreover, it should be reported to the Ethics Committee of Sun Yat-Sen Memorial Hospital of Sun Yat-Sen University within 24 h. The investigator will have to document this serious adverse event and take necessary measures to ensure the participants' safety and interests. It should also be timely reported to the drug administration according to the legislation. At the same time, it should be notified to the investigator involved in the same clinical trials. If it is confirmed as a serious adverse event related to the experimental drug, the investigator will bear the rescue and treatment cost and the corresponding economic compensation.

The investigator must fill in the "serious adverse events report form". The time, the treatment and to whom the SAEs are reported should be recorded in the original data.

14. Concomitant medication

To avoid possible interference from other traditional Chinese medicine, recent therapy (one month prior to and during the IVF/ICSI process) for infertility with TCM will be considered as protocol violation.

The following medications will be allowed during this study, and concomitant medication should be recorded.

(1) Anti-diabetic agents and anti-hypertension agents.

(2) Folic acid supplement aimed at preventing neural tubal defect.

(3) For patients with abnormal bleeding/prolonged amenorrhea, progestin, micronized progesterone, or dydrogesterone.

(4) During controlled ovarian stimulation, human menopausal gonadotropin will be allowed to use in patients with slow E_2 increase or follicles development.

(5) For patients with moderate or severe OHSS, routine clinical treatment, such as fluid infusion, albumin infusion, aspirin, or preventive antibiotics, will be used.
(6) For patients with threatening abortion, an extra dose of progesterone will be allowed to use. Concomitant medication will be recorded.
(7) For patients with pregnancy complications, clinical standard care will be performed. Concomitant medication will be recorded.

15. Monitoring

15.1 Data and Safety Monitoring Board

The clinical trial management office will review and interpret data generated from the study and review the protocol's revisions before their implementation. Its primary objectives are to ensure the safety of study subjects and the integrity of the research data. The office will advise on research design issues, data quality and analysis, and research participant protections for the study. The office will hold regular conference calls to review the protocol for ethical and safety standards, monitor the trials' safety, monitor the data's integrity for original study design, and provide advice on study conduct. The office will review the trial's progress, adjudicate adverse events, and decide on any premature closure of the study. The board will coordinate the call and provide study updates before the call via email.

Table 8. Data and Safety Monitoring Board

Name	Affiliation	Email
Zhaosi Xu	Guilin University of Electronic Technology	zhaosi.xu@jeeyor.com
Xiaoli Chen	Sun Yat-Sen Memorial Hospital of Sun Yat-Sen University	gzxiaolichen@163.com
Jiewen Zhou	Guangzhou University of Chinese Medicine	zhoujiewen0808@163.com

15.2 Ethics

Ethics approval has been sought from the Ethics Committee at Sun Yat-Sen Memorial Hospital, with an ethic approval file entitled: "2017 Reproduction Ethnic Approval No.2" (in Chinese, 2017 生殖伦审字第(02)号). Ethics approval will be obtained from each participating center.

16. Data handling and record-keeping

Prof. Heping Zhang at Yale University will oversee the data collection and management (including quality assurance/compliance measures) team consisting of investigators from Sun Yat-Sen Memorial Hospital.

16.1 Data entry and electronic case report form (eCRF)

The trial investigators must go through Good Clinical Practice (GCP) training and understand the protocol and relevant information in advance adequately. The protocol will be executed strictly, and clinical trial drugs will be provided to enrolled subjects after screening.

Clinical research coordinator (CRC) is responsible for entering the data of CRF into the database. All data required on the eCRF must be recorded. Each blank of the eCRF must be completed, and all items should be filled in. If the item is "not done", then select "ND". Double data entry is performed by two independent CRCs separately to make two databases, which will be compared by the data manager to generate a list of inconsistent data. Any inconsistencies between the two databases are identified by the data manager, and queried via the eCRF system until final settlement.

16.2 Data quality control and query management

Data will be verified by independent statisticians to generate the data query list according to the data verification plan (DVP). Then clinical research associate (CRA)

asks the corresponding investigator to answer the queries in the form, after which the form with answers will be given back to the data manager. The database will be revised based on these forms.

16.3 Data security

The database managers, Dr. Yu Li, biostatistician Prof. Heping Zhang, and project leader Prof. Dongzi Yang, will be in charge of the eCRF records, and are responsible for the assignment of the jurisdiction to the users. The database managers have the highest jurisdiction to manage and monitor the data and actions. The users of each sub-center will be allowed to enter their patients' information and study results in their center. The database managers take the responsibility to decide which data could be disclosed to the public. Patients' information that will or may lead to identity recognition, includes but not limited to name, address, medical card number, telephone numbers, will be critically protected and will never be allowed to be disclosed.

In addition to the internal safeguards built into the computerized system, external safeguards will be implemented. Data will be stored at the servers housed at Sun Yat-Sen Memorial Hospital with access overseen by Prof. Heping Zhang. Records will be regularly backed up, and record logs are maintained to prevent a catastrophic loss and ensure the data's quality and integrity.

16.4 Audit

Audit will be performed during the whole trial process to ensure the data quality. The investigator team will compare data in the database against medical record in the hospital information system (HIS). Identified errors will be resolved between the DCC and clinical sites. The visits will assure data quality and patient protection.

16.5 Medical Coding

Adverse events are coded using MedDRA 21.0 (or higher version).

17. Publication policy

It is anticipated, there will be up to 14 authors in the final manuscript. Yu Li will be the first author. Dongzi Yang and Heping Zhang will be the last two author. The other authors' order for the participating sites will be based upon subject recruitment, data accuracy, and promptness of data report and will start at the position 2 and go to position n-2. Each site's PI will be responsible for documenting the contributions to the study of that site's authors. We encourage the site investigators to establish the second hypothesis and have publications by sharing these data under the publication committee's supervision.

18. Acknowledgment section

The acknowledgment section will include other investigators and study personnel who contributed substantially to the study by site and members of the advisory board and Data Safety Monitoring Board. The designation will list the initials of the individual, followed by their highest degree. Significant contributions include but are not limited to protocol review, initiation and participation at each site, subject recruitment and enrollment, study conduct, data analysis, and manuscript preparation.

19. Protocol revision history

The Application of Zishen Yutai Pill in Aged Women Undergoing IVF-ET. After the initial version was prepared by the protocol committee, this protocol underwent two major revisions by the investigators.

**Version**	**1**
**Date written**	**December 26, 2016**
**Date approved**	**January 12, 2017**
**Version**	**2**
**Date written**	**August 22, 2018**

**Date approved**	**September 20, 2018**
**Version**	**3**
**Date written**	**May 24, 2019**
**Date approved**	**May 30, 2019**

20. References

1. Mathews TJ, Hamilton BE. First Births to Older Women Continue to Rise. NCHS Data Brief (2014) 152:1-8.

2. Bayrampour H, Heaman M, Duncan KA, Tough S. Advanced Maternal Age and Risk Perception: A Qualitative Study. BMC Pregnancy Childbirth (2012) 12:100. doi: 10.1186/1471-2393-12-100.

3. Laopaiboon M, Lumbiganon P, Intarut N, Mori R, Ganchimeg T, Vogel JP, et al. Advanced Maternal Age and Pregnancy Outcomes: A Multicountry Assessment. BJOG (2014) 121 Suppl 1:49-56. doi: 10.1111/1471-0528.12659.

4. Hoorens S, Gallo F, Cave JA, Grant JC. Can assisted reproductive technologies help to offset population ageing? An assessment of the demographic and economic impact of ART in Denmark and UK. Hum Reprod. (2007) 22(9):2471-5. doi: 10.1093/humrep/dem181.

5. American College of Obstetricians and Gynecologists Committee on Gynecologic Practice and Practice Committee. Female age-related fertility decline. Committee Opinion No. 589. Fertil Steril. 2014 101(3):633-4. doi: 10.1016/j.fertnstert.2013.12.032.

6. Huang S-T, Chen AP-C. Traditional Chinese Medicine and Infertility. Curr Opin Obstet Gynecol (2008) 20(3):211-5. doi: 10.1097/GCO.0b013e3282f88e22.

7. Gao Q, Han L, Li X, Cai X. Traditional Chinese Medicine, the Zishen Yutai Pill, Ameliorates Precocious Endometrial Maturation Induced by Controlled Ovarian Hyperstimulation and Improves Uterine Receptivity via Upregulation of HOXA10. Evid Based Complement Alternat Med. (2015) 2015:317586. doi: 10.1155/2015/317586.

8. Zhang Y, Yan W, Ge PF, Li Y, Ye Q. Study on prevention effect of Zishen Yutai pill combined with progesterone for threatened abortion in rats. Asian Pac J Trop Med. (2016) 9(6):577-81. doi: 10.1016/j.apjtm.2016.04.002.
9. Xu H, Luo Y. The application Zishen Yutai Pill in the ovulation protocol of polycystic ovary syndrome. Chin J Information Tradit Chin Med. (2008) 15(8):68.
10. Zhu WJ, Li XM, Chen XM, Zhang L. Effect of Zishen Yutai pill on embryo implantation rate in patients undergoing fertilization embryo transfer in vitro. Chin J Integr Med. (2002) 22(10): 729-737. doi: 10.3321/j.issn:1003-5370.2002.10.002.
11. Maheshwari, A., E.A. Raja, S. Bhattacharya. Obstetric and perinatal outcomes after either fresh or thawed frozen embryo transfer: an analysis of 112,432 singleton pregnancies recorded in the Human Fertilisation and Embryology Authority anonymized dataset. Fertil Steril, 2016. 106(7):1703-1708.
12. Alpha Scientists in Reproductive Medicine and ESHRE Special Interest Group of Embryology. The Istanbul consensus workshop on embryo assessment: proceedings of an expert meeting. Hum Reprod. (2011) 6:1270-83. doi: 10.1016/j.rbmo.2011.02.001
13. Gardner DK, Balaban B. Assessment of Human Embryo Development Using Morphological Criteria in an Era of Time-Lapse, Algorithms and 'Omics': Is Looking Good Still Important? Mol Hum Reprod (2016) 22(10):704-18. doi: 10.1093/molehr/gaw057
14. Shi Y, Wei D, Liang X, Sun Y, Liu J, Cao Y, et al. Live Birth after Fresh Embryo Transfer Vs Elective Embryo Cryopreservation/Frozen Embryo Transfer in Women with Polycystic Ovary Syndrome Undergoing IVF (Frefro-Pcos): Study Protocol for a Multicenter, Prospective, Randomized Controlled Clinical Trial. Trials (2014) 15:154. doi: 10.1186/1745-6215-15-154.

Supplementaty Note 2. Protocol revision history and major changes

Protocol revision history and major changes

1 Protocol revision history

The Application of Zishen Yutai Pill in Aged Women Undergoing IVF-ET. After the initial version was prepared by the protocol committee, this protocol underwent two major revisions by the investigators.

**Version**	**1**
**Date written**	**December 26, 2016**
**Date approved**	**January 12, 2017**
**Version**	**2**
**Date written**	**August 22, 2018**
**Date approved**	**September 20, 2018**
**Version**	**3**
**Date written**	**May 24, 2019**
**Date approved**	**May 30, 2019**

2 Summary of Major Revisions

Revisions from Ver 1 to Version 2

No	Section	Major changes	Note
1	1.2	Study sites had been determined as follows,<br>Sun Yat-Sen Memorial Hospital of Sun Yat-Sen University<br>The Guangxi Zhuang Autonomous Region Health and Family Planning Commission Reproductive Center<br>The Fourth Hospital of Shijiazhuang<br>Reproductive and Genetic Hospital of CITIC-XIANGYA<br>The First Hospital of Lanzhou University<br>Yantai Yuhuangding Hospital<br>Northwest Women's and Children's Hospital<br>West China Second University Hospital of Sichuan University<br>Yinchuan Maternal and Child Healthcare Hospital<br>The First Affiliated Hospital of Xinjiang Medical University	
2	4.1	Inclusion criteria had been changed from "BMI<30 kg/m^2" to "BMI<28 kg/m^2"	In our previous research, we applied the WHO obesity cutoff (BMI < 30 kg/m^2). To align with the 2016 Chinese Guidelines on Medical Nutritional Therapy for Overweight/Obesity (DOI: 10.3969/j.issn.1672-7851.2016.09.003), which define overweight/obesity as 24–28 kg/m^2, the criterion was revised to better reflect the Chinese population.
3	6.4	ET visit (4). From "Information about embryo development, embryo transfer information" to "Information about semen collection, embryo development, embryo transfer information"	To ensure complete recording of male partner information and fertilization parameters.

4	12.2	Sample size estimation, from 644 to 1466.	The initial estimate (644 participants) was based on retrospective single-center data (ZYP = 48%, placebo = 35%). After unblinding results of AMA women from our previous randomized clinical trial (DOI: 10.1097/AOG.0000000000004658), the sample size was recalculated using empirically derived live birth rates (ZYP = 42%, placebo = 33%), requiring 1,466 participants to achieve 80% power at $\alpha = 0.05$, accounting for a 38% attrition rate (including both dropout and cancellation).

Revisions from Ver 2 to Ver 3

No	Section	Major changes	Note
1	1.2	Study sites had been determined as follows,<br>Sun Yat-Sen Memorial Hospital of Sun Yat-Sen University<br>Women and Children's Hospital of Chongqing Medical University<br>Tangdu Hospital, the Fourth Military Medical University<br>The First Affiliated Hospital of Zhengzhou University<br>The First Hospital of Lanzhou University<br>Liuzhou Maternity and Child Healthcare Hospital<br>West China Second University Hospital of Sichuan University<br>The Affiliated Suzhou Hospital of Nanjing Medical University<br>Northwest Women’s and Children's Hospital<br>Reproductive and Genetic Hospital of CITIC-Xiangya	

		The Third Affiliated Hospital of Zhengzhou University<br>Hospital for Reproduction Medicine Affiliated to Shandong University	
2	3/10/12.3	Primary outcome(s) changed from “clinical pregnancy rate and live birth rate” to “fresh cycle live birth rate”.	The co-primary outcomes in Version 2 were revised to a single primary outcome, i.e., fresh cycle live birth rate. The specification of “fresh cycle” was added to avoid ambiguity and ensure consistency in analysis.
3	5.3.3	High-quality embryos definition was added.	To standardize the evaluation of embryo quality across centers.

Supplementaty Note 3 : Informed Consent and translation

Informed Consent Form

Trial Name

Zishen Yutai Pills in infertile women with advanced maternal age undergoing IVF/ICSI-ET: A multicenter, prospective, double-blinded, randomized placebo-controlled trial

Lead Affiliation: Sun Yat-Sen Memorial Hospital of Sun Yat-Sen University

Site:

Inform Consent

Trial Name

Zishen Yutai Pills in infertile women with advanced maternal age undergoing IVF/ICSI-ET: A multicenter, prospective, double-blinded, randomized placebo-controlled trial

We sincerely invite you to participate in the above-named study. You are required to decide participating or not. Please read the following information carefully before you made up your mind. You can ask any questions with your doctor.

Why is this study being done?

Zishen Yutai Pill (ZYP) is a formula developed by Professor Luo Yuankai, a renowned traditional Chinese medicine (TCM) practitioner from Guangdong Province and a professor at Guangzhou University of Chinese Medicine. ZYP has been used for decades to prevent miscarriages and treat infertility due to *kidney* deficiency. It has won the second prize for Scientific and Technological Achievement Reward from the Ministry of Health and has been designated as a national protected traditional Chinese medicine.

Clinical observations by Xu Hai'ou and others have shown that using ZYP in polycystic ovary syndrome (PCOS) ovulation induction protocols, in combination with clomiphene, an ovulation-inducing drug , would increase ovulation and pregnancy rates while reducing miscarriage rates compared with using clomiphene alone. Additionally, clinical observations by Zhu Wenjie and others have shown that taking ZYP during the luteal phase could effectively improve embryo implantation rates in patients undergoing in vitro fertilization-embryo transfer (IVF-ET). Adverse assisted reproductive outcomes in advanced maternal age (AMA) undergoing IVF-ET are global challenges. And AMA possibly necessitate overall physical adjustment, because reproductive endocrine function generally declines with age. It still lacks prospective clinical researches with large sample size that whether taking ZYP during ART for AMA can

improve oocyte retrieval numbers, embryo quality and implantation rates through traditional Chinese medicine mechanisms, thereby enhancing clinical pregnancy and live birth rates.

This study aims to evaluate the effectiveness of using ZYP in AMA with infertility undergoing IVF/ICSI-ET, particularly its impact on the ovulation induction process and pregnancy outcomes.

Several reproductive centers in China will participate in this study, encompassing 1,466 women.

This project has received funding from the Guangdong Provincial Administration of Traditional Chinese Medicine and the Guangzhou Municipal Science and Technology Plan Research Project.

Who should be in this study?

You will be included in this study if you have the following:

(1) Infertile women aged between 35 and 42 years old.

(2) Intend to undergo IVF/ICSI-ET (GnRH-a long protocol or GnRH-ant protocol).

(3) BMI<28 kg/m^2.

(4) Bilateral ovaries exist.

(5) Patients who voluntarily sign the informed consent and agreed to be followed up as required by the study protocol.

You will not be included in this study if you have the following:

(1) Recurrent implantation failure (previous three times or more IVF/ICSI-ET failure).

(2) Adenomyosis, the uterine cavity line constricted by uterine fibroids.

(3) Untreated bilateral hydrosalpinx.

(4) Endometrial diseases that have not been cured.

(5) Known diseases that are not suitable for undergoing ART or at the present not suitable for pregnancy;

(6) Recent therapy (within one month) for infertility with TCM.

What do you need to do to participate in this study?

If you are included in this study, all the treatments you receive will be the standard clinical procedures, identical to regular "in vitro fertilization" except that in this study, there will be two groups of patients. In the IVF assisted reproduction treatment, you will be randomly and double-blindly assigned to one of the groups in a 1:1 ratio. One group will receive the study drug and the other group receive the placebo. Please ensure to inform your doctor of any other medications you are currently using or plan to use during the study. During the study, you should not use any other traditional Chinese medicines to treat infertility. If other treatments are needed, please contact your doctor in advance for proper medical guidance. If you achieve a clinical pregnancy, the follow-up during the pregnancy will be more frequent than regular patients, and researchers will call to ask about any complications during the pregnancy. After your delivery, researchers will follow up to learn about the delivery information (mode of delivery, any pregnancy complications) and neonate information (newborn's gender, weight, height, any birth defects, whether admitted to NICU within the first month, or any stillbirths). Blood samples of 5ml (in anticoagulant tubes) will be taken on the initiation day, oocyte retrieval day, and 14 days after embryo transfer. Additionally, semen parameters (concentration, volume, sperm motility, fertilization method) will be collected. If you undergo a frozen embryo transfer after canceling a cycle, your first frozen embryo cycle will be monitored.

How long will I be in the study?

The treatment process requires 1 to 2 months. If you achieve a clinical pregnancy, the follow-up during pregnancy and postpartum will continue for about 11 months. Therefore, from your inclusion in the study to the end of the trial, it will take approximately 12 to 14 months.

What adverse (bad) effects may happen to me by participating in the study?

Participating in this study does not increase any additional risks compared with patients undergoing usual "in vitro fertilization" treatments. The potential risks and adverse

reactions associated with standard IVF techniques, such as embryo transfer, embryo freezing, etc., are detailed in the informed consent documents for these procedures. Below is a table summarizing these risks:

Procedures and events	Risks and discomfort
Controlled ovarian hyperstimulation (COH)	Frequent subcutaneous injection, frequent venipuncture, frequent transvaginal ultrasound scan. Supra-physiologic E_2 may increase the risk of cancer ovary torsion or ovary rupture.
Ovarian hyperstimulation syndrome (OHSS)	Massive enlargement of the ovaries, ascites, bloating, nausea, and vomiting. Severe cases may have thoracic edema, breathing difficulties, oliguria, even anuria, and may require hospitalization, medication, or puncture drainage. A very severe case may suffer from thrombosis, damage to the liver or renal function, and even death.
Oocyte retrieval	Anesthesia accident, pelvic organ injury, intra-abdominal hemorrhage, puncture site hemorrhage, in serious case surgery or transfusion may be needed, infection.
ICSI	Microinjection may injure an oocyte, pass an unknown disease gene to the next generation.
Embryo transfer	Infection.
Embryo frozen and thaw	Embryotic development arrest. The survival rate of thawed embryos is 95%.
Standard venipuncture for blood work	Slight pain, ecchymosis at the site of puncture, infection, or bleeding at the site.
Transvaginal ultrasound	Abdominal or pelvic discomfort.
Ectopic pregnancy	May require medical or surgical treatment. In severe cases, pregnancy site rupture can result in intra-abdominal hemorrhage, even shock, or death if treatment is delayed.
Multiple pregnancies	May require embryo reduction, increase risk of pregnancy complication, fetus abnormalities, and preterm delivery.

Infertility treatment	Anxiety or emotional distress to various degrees.
Zishen Yutai Pill	It has been reported that some patients who took the Zishen Yutai pill suffered from nausea, dry mouth, and constipation that disappeared after drug withdrawal.

Not all patients will experience the aforementioned adverse events.

This study will exclude patients who have contraindications for "in vitro fertilization" or pregnancy. The oocyte retrieval and embryo transfer processes will be performed by qualified and experienced doctors. Each sub-center will inform patients of the researchers' phone numbers or the phone numbers of the resident doctors available 24 hours a day, so that patients can contact us in case of any adverse events. This study will record all adverse events including serious adverse events.

The study will make every effort to minimize harm to participants, but it cannot eliminate the possibility of complications or injuries occurring during the study. In the event of an adverse event, we will provide active treatment, and if it involves a medical dispute, it will be handled according to the hospital's conventional procedures for medical incidents.

Related costs: Participating in this study will not incur any additional costs. The medications used in the study are provided free of charge, and all other tests, medications, and treatments are conventional.

What are the benefits of participating in this study?

You will be randomly assigned to one of the treatment groups, and the final results of the study may indicate that the treatment you receive is more effective or has a lower rate of adverse reactions compared to the other group and other treatment methods.

Can I refuse to be in the study?

Participation in this study is voluntary, and you can refuse to participate. If you decide to participate, you will receive a copy of this information and you will need to sign a written informed consent form. During the study, you can request to withdraw at any

time without any penalty, and your treatment will not be affected.

If you experience adverse reactions during the study that may affect your health, or if you do not comply with the study requirements, the researchers may ask you to withdraw without your consent. If you choose to withdraw from the study before it concludes, you should inform the researchers at the time of your departure.

During your participation, we will keep you informed about medical research developments related to your condition, and you can decide whether to continue participating in the study based on this information.

Confidentiality and privacy

This study will strictly adhere to privacy protection policies. All communications with our institution will be securely stored, and any personally identifiable information will not be disclosed to anyone outside of this research for any reason. Your personal information will not be disclosed even if the results of this study are published.

You have the right to access your personal information and the final results of the study. Your personal information will be protected during data collection, storage, and application processes, including analysis and comparison.

Contact Information for the Study

You have the right to ask any questions about this study. If you have concerns, questions, or consider that you have experienced an adverse reaction, please call this number during work hours __________, and this number after work hours __________ to contact us.

If you have any questions about your rights as a participant, or concerns about the confidentiality of your privacy, you can contact the hospital's ethics committee at this number __________. If you are unable to directly reach the researchers of this study, you can call this number.

Informed Consent Form • Consent Signature Page

Participant Statement

I have read (or the researcher has explained to me) the information related to participating in this study, and I have had ample time to consider whether to participate. The researcher has satisfactorily answered my questions. I agree to participate in this study and consent to the use of data from my medical records by the researchers of this study.

I understand:

1. This is a clinical study aimed to evaluate the effectiveness of using ZYP in AMA undergoing IVF/ICSI-ET, and its impacts on the ovulation induction process and pregnancy outcomes.
2. My identity and any personally identifiable information will be kept confidential.
3. My participation in this study is voluntary, and I can withdraw at any time without any impact on my treatment.
4. I can ask the doctor or researcher any questions about the study.

Signing below indicates that I have understood the above information, have asked questions about any uncertainties, and have received answers. I voluntarily agree to participate in this clinical study.

Participant's signature: Contact information:

Date: Year Month Day

Spouse's signature: Contact information:

Date: Year Month Day

Researcher Statement

I have fully explained and clarified to the participant the purpose of this clinical study, the methods of research, operational procedures, and the potential risks and benefits of participating in this study. I have satisfactorily answered all the participant's questions.

Researcher's signature: Contact information:

Date: Year Month Day

知情同意书

临床研究项目名称：滋肾育胎丸在高龄女性体外受精-胚胎移植治疗中的应用：一项多中心、前瞻性、随机、对照、双盲的临床研究.

临床研究申办单位：中山大学孙逸仙纪念医院

参研单位：

知情同意书•知情告知页

项目名称：滋肾育胎丸在高龄女性体外受精-胚胎移植治疗中的应用：一项多中心、前瞻性、随机、对照、双盲的临床研究.

我们诚邀您参加这项临床研究，研究题目如上，您需要决定是否愿意参加，做出决定前请仔细阅读下文内容，您有任何问题都可以询问医生。

为何要进行该项研究?

滋肾育胎丸是广东省名老中医、广州中医药大学罗元恺教授几十年用于防治流产和肾虚不孕症的验方。获得卫生部科技成果二等奖，被评为国家中药保护品种。

许海鸥等临床观察滋肾育胎丸在多囊卵巢综合征促排卵方案中的应用，提示滋肾育胎丸与促排卵药氯米芬同用,较单纯西药促排卵组提高了排卵率、妊娠率,并降低了流产率。而朱文杰等临床观察黄体期口服滋肾育胎丸对体外受精-胚胎移植患者胚胎种植率的影响，结果发现黄体期口服滋肾育胎丸能有效地提高胚胎种植率。高龄女性行体外受精与胚胎移植的助孕结局不良是国内外生殖医学领域的难题，而高龄女性的生殖内分泌功能整体衰退，可能更需要进行整体体质的调节。在高龄不孕女性助孕过程中加用滋肾育胎丸是否能通过中医调节的机制，提高获卵数、提高胚胎的质量及种植率，从而有利于提高最终高龄女性的临床妊娠率及活产率，尚缺乏大样本的前瞻性临床研究。

本项研究的目的是评价接受 IVF/ICSI-ET 治疗的高龄不孕女性，使用滋肾育胎丸对 IVF-ET 促排卵过程及妊娠结局影响的有效性。

全国将有多家生殖中心参加本研究，共 1466 例女性将被纳入研究。

本课题已获得广东省中医药局及广州市科技计划研究项目的经费资助。

哪些患者将会被参加该项研究？

您被邀请参加本研究，是因为您有下述情况：

1. 35≤年龄≤42 周岁的不孕妇女；
2. 拟行体外受精-胚胎移植助孕者（长方案及拮抗剂方案）；
3. BMI＜28kg/m^2；
4. 双侧卵巢存在。
5. 自愿签署知情同意书，并同意按照研究方案的要求接受随访的患者。

如果您有下述情况，您将不能参加该项研究：

1. 反复种植失败（既往 3 次及以上 IVF/ICSI-ET 失败者）；
2. 子宫腺肌症、子宫肌瘤压迫宫腔线；
3. 未处理的双侧输卵管积水；
4. 未治愈的子宫内膜疾病；
5. 患有不适合目前进行辅助生殖技术或者不适合目前妊娠的疾病；
6. 近一个月 (30 天) 内服用治疗不孕症相关中药或中成药的患者。

参加该项研究，您需要干什么？

如果您被纳入本研究，您所接受的所有治疗都是临床上常规进行的治疗措施，与通常"试管婴儿"唯一不同的是在该项研究中将有 2 组患者，在 IVF 助孕治疗中您将会按照 1：1 的比例被随机双盲分配到两组中的一组，一组服用药物，一组服用安慰剂。请您务必告知您的医生您目前正在使用以及在研究期间使用的任何其它药物。研究期间，请您不要使用任何其他治疗不孕症的中药，如需其他治疗，请事先与您的医生联系，以获得正规医学指导。如果您获得了临床妊娠，妊娠期间的随访比普通患者更加密切，研究人员会通过电话询问您妊娠期并发症的相关信息。在您分娩后，研究人员会随访了解分娩（分娩方式、是否有妊娠合并症）及新生儿健康方面(新生儿性别、体重、身长、是否存在出生缺陷、近一月内是否转入 NICU、是否出现死产）的信息。在启动日、取卵日、移植后 14 天均留取血标本 5ml（抗凝管）。同时，会对男性精液处理后情况（浓度、量、精子活动力、受精方式）进行收集。若您取消周期后进行了冻胚移植，则会对您第一次冻胚周期的情况进行访视。

参加该项研究需要多久？

治疗过程需 1~2 个月，如果您获得了临床妊娠，对妊娠期以及产后的随访会持续约 11 个月。因此从您被纳入该研究至研究结束，大约需要 12~14 个月时间。

参加该项研究会有什么危险和副反应？

与通常的接受"试管婴儿"的患者比较，参加该项研究不会增加额外风险。通常的"试管婴儿"技术中可能的风险和不良反应都在体外受精胚胎移植、胚胎冷冻等的知情同意书中有详细的说明。下面的表格是对这些风险总结：

步骤或事件	不适或风险
促排卵过程	频繁的皮下注射、抽血、B 超；超过生理水平的雌激素可能增加癌症风险；卵巢体积增大导致卵巢扭转或破裂
卵巢过度刺激综合征(OHSS)	卵巢明显增大，腹水，腹胀，恶心呕吐，少数重度患者可出现胸水和呼吸困难，少尿甚至无尿，可能需要住院、用药甚至胸腹水引流，极个别情况可能发生血栓、肝肾功能损害甚至死亡
取卵	麻醉意外，盆腔器官损伤，感染，腹腔内出血，穿刺部位出血，个别严重情况可能需要手术或者输血治疗
单精子胞浆注射（ICSI）	显微注射可能损伤卵子，使未知的致病基因传给下一代
胚胎移植	感染
胚胎冷冻和解冻	胚胎发育停滞，目前本中心解冻胚胎的存活率是 95%
抽血化验	轻微疼痛，穿刺部位淤青、感染或出血
经阴道 B 超检查	腹部或盆腔部位不适感
异位妊娠	可能需要药物或手术治疗，个别严重情况发生妊娠部位破裂，导致腹腔内出血，如果延误治疗可能导致休克甚至死亡
多胎妊娠	可能需要减胎手术，妊娠期并发症，胎儿异常及早产的风险增加
不孕症治疗	焦虑、情绪低落等
滋肾育胎丸	据报道，有患者服用滋肾育胎丸之后，会出现恶心反胃、口干、便秘等表现，停药后缓解

并非所有患者都会出现上述不良事件。

本研究将排除存在“试管婴儿”或者妊娠禁忌症的患者。取卵和胚胎移植过程将由有资质而且有经验的医生完成。每个分中心都将告知患者研究者电话或者值 24 小时班的住院医师的电话，一旦患者发生不良事件可以联系到我们。本研究将记录所有患者发生的不良事件，包括严重不良事件。

本研究将尽最大努力减少对参与者造成的损伤，但是不能排除参加本研究会发生并发症或者损伤的可能。发生不良事件时，我们会对您进行积极的治疗，如果涉及医疗纠纷，则按医院常规的医疗事件进行处理。

相关费用：参加该研究不会额外增加任何费用，研究所用的药物是免费的，其他的检查、用药及治疗均为常规。

参加本研究会有什么好处？

您将被随机分配到任一治疗组，最后的研究结果可能表明您接受的治疗比另外 1 组及其他治疗方法更有效或者不良反应的发生率更低。

您可以拒绝参加该项研究吗？

参加该项研究是自愿的，您可以拒绝参加。如果您决定参与该项研究，您将可以留1份本资料，您需要签署书面的知情同意书。在研究期间您可以要求退出本研究，您的退出将不会受到任何惩罚，您的治疗不会受到任何影响。

如果您在参加研究的过程中发生了不良反应而继续参加本研究可能会影响您的健康，或者您没有按照本研究的要求去做，研究者有可能在未获得您同意的情况下要求您退出要求。如果您在研究结束之前退出研究，您需要在您退出的时候告知研究者。

在您参加该研究期间，我们会告知您与您所患疾病相关的医学研究进展，了解这些进展后，您可以决定是否继续参加本研究。

隐私与保密

本研究将严格执行隐私保护政策，所有与本单位的通信联系都将被妥善保管，任何能识别您的个人信息都不会因为任何理由而泄露给本研究以外的任何人。即使本研究的结果被发表，您的个人信息也不会被公开。

您有权利了解您的个人信息和最终的研究结果。您的个人信息在数据收集、保存及应用（包括分析和比较）的过程中都将受到隐私保护。

该研究的联系方式

您有权利询问关于本研究的任何问题。如果您有疑问、顾虑或者您认为自己发生了不良反应，请打这个电话__________，非工作时间请打这个电话__________联系我们。

如果您对于参加本研究您所具有的权利有任何疑问，或者对于您的隐私的保密有顾虑，您可以联系医院的伦理委员会，电话是__________，如果您未能直接联系到本研究的研究人员，您可以拨打这个电话。

知情同意书•同意签字页

受试者声明

我已经阅读了（或者研究者已经向我介绍了）参加本研究的相关信息，我有充足的时间考虑是否参加该研究。研究者对我的疑问给予了满意的回答。我同意参加该研究，同意该项研究的研究人员使用我的病历记录中的数据。

我知道:

1.这是一项临床研究，目的是比较滋肾育胎丸用于体外受精胚胎移植的高龄女性对促排卵过程及妊娠结局影响的有效性。

2.我的身份及可以识别我的信息都将被保密。

3.我参加这项研究是自愿的，我可以退出研究，而我的治疗将不受到任何影响。

4.我可以向医生或者研究者询问任何关于该研究的信息。

在下面签名表示我已经理解了上述信息，已经对疑问之处做了询问并得到了回答，我自愿参加该临床研究。

受试者签字：________________　　　　　　联系方式：_______________________

日期：_____年_____月____日

受试者配偶签名：____________　　　　　　联系方式：______________________

日期：_____年_____月____日

研究者声明

我本人已向该受试者充分解释和说明了本临床研究的目的、研究方法、操作流程以及受试者参加该研究可能存在的风险和潜在的利益，并满意地回答了受试者的所有有关问题。

研究者签字：_________________　　　　　　联系方式：____________________

日期：_______年______月____日

Supplementaty Note 4: List of participating sites, ethics committee and approval number

List of participating sites, ethics committee and approval number

No.	Site Name	Ethics Committee Name	Ethical Approval Number
1	Sun Yat-Sen Memorial Hospital of Sun Yat-Sen University	Ethics Committee of Reproductive Medicine, Sun Yat-sen Memorial Hospital of Sun Yat-sen University,	2017 Reproduction Ethnic Approval No.2
2	Women and Children's Hospital of Chongqing Medical University	Clinical Application and Ethics Committee, Human Assisted Reproductive Technology of Women and Children's Hospital of Chongqing Medical University	2019 Ethnic (Research) -01
3	Tangdu Hospital, the Fourth Military Medical University	IEC of Institution for National Drug Clinical Trials, Tangdu Hospital, Fourth Military Medical University	K201909-06
4	The First Affiliated Hospital of Zhengzhou University	The Research and Clinical Trial Ethics Committee of the First Affiliated Hospital of Zhengzhou University	2019-178
5	The First Hospital of Lanzhou University	Ethics Committee of First Hospital of Lanzhou University	LDYYSZLL 2019-17
6	Liuzhou Maternity and Child Healthcare Hospital	Ethical Committee for Drug Clinical Trials of Liuzhou Maternity and Child Healthcare Hospital	PJ2017012
7	West China Second University Hospital, Sichuan University	Medical Ethics Committee of West China Second University Hospital, Sichuan University	2019-066
8	The Affiliated Suzhou Hospital of Nanjing	The Ethics Committee of the Affiliated	IEC-C-008-A07-V1.0

	Medical University	Suzhou Hospital of Nanjing Medical University	
9	Northwest Women’s and Children's Hospital	Clinical Application Ethics Committee of Human Assisted Reproduction Technology of Northwest Women and Children's Hospital	2019001
10	Reproductive and Genetic Hospital of CITIC-Xiangya	Ethics Committee of Reproductive and Genetic Hospital of CITIC-Xiangya	LL-SC-2019-012
11	The Third Affiliated Hospital of Zhengzhou University	Ethics Committee for Drug Clinical Trials of the Third Affiliated Hospital of Zhengzhou University	IEC-C-008-A07
12	Hospital for Reproduction Medicine Affiliated to Shandong University	Ethics Committee of Hospital for Reproductive Medicine Affiliated to Shandong University	2020-98

Supplementary Note 5: Study Drug

Content

1. Study drug and placebo 2
1.1 Study drug, Zishen Yutai Pill (ZYP) 2
1.2 Placebo 4
1.3 Package and label of study drug 4
2. The method of fingerprint analysis of Zishen Yutai Pill 6
2.1 Chemicals and drugs 6
2.2 Preparation of sample 6
2.3 UHPLC-CAD fingerprint analysis 6

1. Study drug and placebo

1.1 Study drug, Zishen Yutai Pill (ZYP)

(1) Manufacture of ZYP

The ZYP is manufactured by Baiyunshan Zhongyi Pharmaceutical Co. Ltd., containing 15 herbal drugs (**Table S1**). The manufacturer of ZYP complies with the relevant requirements of law of China's Drug Administration and Good Manufacturing Practice (GMP), with approval from the China National Medical Products Administration (Permit No.Z44020008).

(2) Appearance, odor and taste of ZYP

ZYP is a black-coated concentrated water-honeyed pill. Upon removal of the coating, it reveals a deep brown color, with a faintly aromatic odor and a slightly bitter taste.

Table S1. Herbal drugs used in Zishen Yutai Pill (ZYP) and the origins and medicinal part

Medicine material	Origin of natural medicine	Standard prescription amount/g
Cuscutae Semen	Ripe dried seed of *Cuscuta Chinensis* Lam.	800
Ginseng Radix et Rhizoma	Dried root and rhizome of *Panax ginseng* C. A. Mey.	50
Dipsaci Radix	Dried root of *Dipsacus asper* Wall. ex DC.	480
Taxilli Herba	Dried leafy stem and branch of *Taxillus chinensis* (DC.) Danser	480
Eucommiae Cortex	Dried bark of *Eucommia ulmoides* Oilv.	290
Morindae Officinalis Radix	Dried root of *Marinda officinalis* How	190
Cervi Cornu Degelatinatum	Residue after water extraction of ossified antler of *Cervus nippon* Temminck	140
Codonopsis Radix	Dried root of *Codonopsis pilosula* (Franch.) Nannf.	580
Atractylodis Macrocephalae Rhizoma	Dried rhizome of *Atractylodes macrocephala* Koidz.	240
Asini Corii Colla	Solid glue prepared by stewing and concentrating from the hide of *Equus asinus* L.	30
Lycii Fructus	Dried ripe fruit of *Lycium barbarum* L.	190
Rehmanniae Radix Praeparata	Steamed and dried root of *Rehmannia glutinosa* (Gaertn.) DC.	480
Polygoni Multiflori Radix Praeparata	Steamed and dried root of *Polygonum multiflorum* Thunb.	240
Artemisiae Argyi Folium	Dried leaf of *Artemisia argyi* Lévl. et Vant.	140
Amomi Fructus	Dried fruit of *Amomum villosum* Lour.	70

Note: The standard prescription yields approximately 1200 g of pills. According to the clinical protocol, ZYP is given 5g each time, three times daily, that is at a dose of 15 g/d.

1.2 Placebo

The placebo is manufactured by Baiyunshan Zhongyi Pharmaceutical Co. Ltd.

Composition: Pregelatinized starch, microcrystalline cellulose, black iron oxide, refined honey, dextrin.

Manufacturing process: Pregelatinized starch, microcrystalline cellulose, and black iron oxide are mixed, crushed, and sieved. The refined honey is added to the mixed powder to make the wet pill with the required size. After drying, it is coated with a mixture of black iron oxide and talc powder, 3% dextrin solution, 75% ethanol solution, and refined honey. Finally, eligible pills are polished using Chinese insect wax, selected, and packaged.

The placebo pills have similar characteristics to ZYP, including color, appearance, package, label (See **Figure S1**).

1.3 Package and label of study drug

Both the ZYP and the placebo are provided in the same label and package (written in Chinese). Below is the translation of label and comparison of ZYP and the placebo.

Drug No.:

The Application of Zishen Yutai Pill in Aged Women Undergoing IVF-ET

（Clinical Use Only）　　　　Dosage of one week

【**Action and Use**】Tonify *Kidney* and *Spleen*, invigorates *Qi* that nourishes *Blood*, placate the fetus and strengthens the body. Used in pregnancy loss due to deficiency of *Kidney* and *Spleen*, debility of *Chong* and *Ren* (treatment and prevention of threatened miscarriage and spontaneous miscarriage)。

【**Package**】5 g/package.

【**Dosage and Administration**】Administered orally with honey water or dilute salt water. 5 g (1 package) each time, tid.

【**Intervention period**】11 weeks.

【**Storage Condition**】Please keep in seal and away from moister.

【**Caution**】Please keep the remaining drug and the package until the end of clinical trial. Stored away from children.

Manufactured by Guangzhou Baiyunshan Zhongyi Pharmaceutical Co. Ltd.

Expired Date: ** Batch No.: **

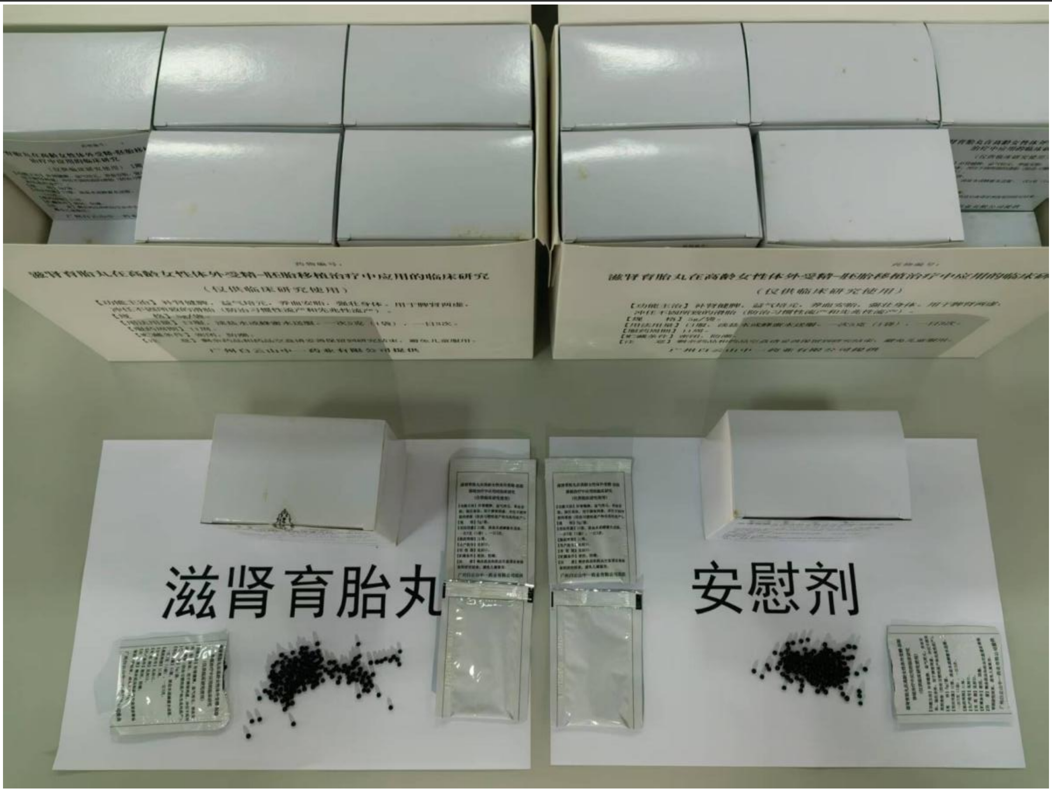

Figure S1. Comparison of ZYP (the left side: 滋肾育胎丸) and the placebo (the right side: 安慰剂)

2. The method of fingerprint analysis of Zishen Yutai Pill

ZYP exhibited similarity of more than 0.82 by ultrahigh performance liquid chromatography-charged aerosol detector (UPLC-CAD), indicating the ingredients of ZYP are stable and controllable according to quality control method as shown in **Figure S2** and **Table S2** (Cao, et al, 2020).

2.1 Chemicals and drugs

Sixteen batches of commercial product ZYP with different lot numbers were provided by Guangzhou Baiyunshan Zhongyi Pharmaceutical Co., Ltd (Guangzhou, Guangdong province, China). HPLC-grade acetonitrile, methanol and formic acid were purchased from Merk (Darmstadt, Germany). The purified water (18.2 MΩ.cm at 25°C) was prepared by a Milli-Q purification system (Millipore, Bedford, MA, USA). Other reagents were all of analytical grade.

2.2 Preparation of sample

An aliquot of 2 g powder sample was weighed and extracted in 20 mL methanol by an ultrasonic cleaner (Branson 5510, 135 W, 42 kHz) for 60 min at room temperature. Then, the extract was centrifuged at 15,800×g for 10 min, and the supernatant was transferred to a HPLC sample vial for chromatographic fingerprint analysis.

2.3 UHPLC-CAD fingerprint analysis

The UHPLC chromatographic fingerprint analysis was performed on DionexUltiMate 3000 rapid separation binary system (Dionex, Thermo Fisher Scientific Inc., USA), equipped with an SRD-3600 degasser, an HPG-3400RS binary pump, a WPS-3000TRS autosampler, a TCC-3000RS column thermostat and a Corona Veo RS charged aerosol detector. The samples were separated on a Hypersil gold C18 (150 mm × 2.1 mm, 1.9 μm, Thermo Fisher) column by gradient elution with the mobile phases of 0.1 % formic acid (v/v) solution (A) and acetonitrile (B). The gradient program was set as follows: 0−10 min, 5% B; 10−20 min, 5–10 % B; 20−40 min, 10–25 % B; 40−60 min, 25–30 % B; 60−75 min, 30–40 % B; 75−90 min, 40–75 % B; 90−120 min, 75−90% B; 120−140 min, 90−100% B; 140−190 min, 100 % B. The flow rate of the mobile phase was kept at 0.4 mL min^{-1}. CAD parameters were as follows: power function, 1.00; data collection rate, 10 Hz; filter, 5.0; evaporation

temperature, 35℃. The column temperature was set at 40℃ and the injection volume was 4 μL. Data acquisition and analysis were carried out using Thermo ScientificTM Dionex Chromeleon 7.2 SR4 software. Similarity analysis was conducted on Similarity Evaluation System for Chromatographic Fingerprint of Chinese Materia (Version 2012).

Reference

Cao J, et al. Development of a comprehensive method combining UHPLC-CAD fingerprint, multi-components quantitative analysis for quality evaluation of Zishen Yutai Pills: A step towards quality control of Chinese patent medicine. J Pharm Biomed Anal. 2020;191:113570. doi: 10.1016/j.jpba.2020.113570

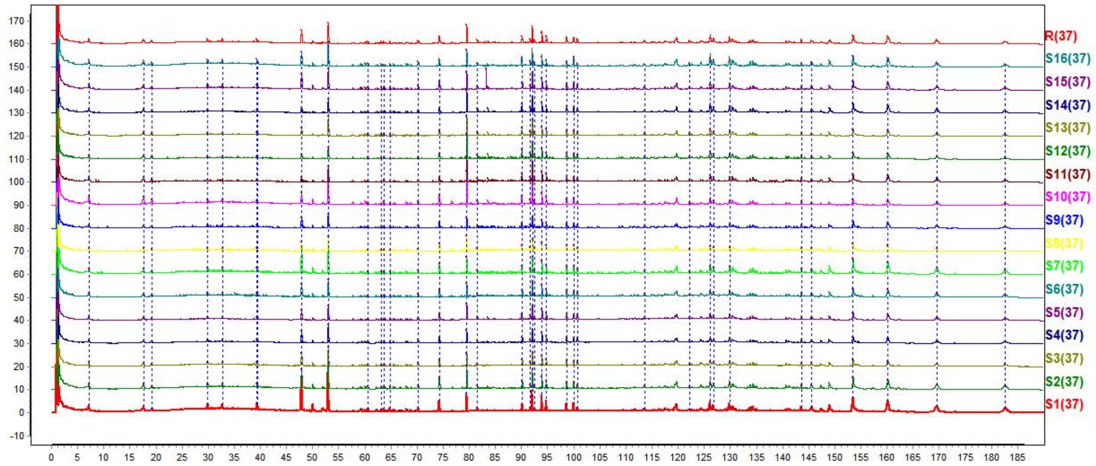

Figure S2. UPLC fingerprint for 16 different batches of ZYP and reference

Table S2. Results of similarity analysis from 16 batches of ZYP Samples

No.	S1	S2	S3	S4	S5	S6	S7	S8	S9	S10	S11	S12	S13	S14	S15	S16	Reference
**S1**	1	0.974	0.973	0.971	0.942	0.965	0.989	0.953	0.978	0.849	0.868	0.825	0.931	0.875	0.973	0.957	0.969
**S2**	0.974	1	0.989	0.989	0.985	0.993	0.983	0.993	0.989	0.898	0.932	0.911	0.967	0.935	0.996	0.975	0.994
**S3**	0.973	0.989	1	0.999	0.982	0.987	0.971	0.980	0.986	0.917	0.921	0.906	0.973	0.915	0.993	0.970	0.991
**S4**	0.971	0.989	0.999	1	0.982	0.988	0.97	0.979	0.987	0.91	0.921	0.905	0.971	0.918	0.991	0.969	0.990
**S5**	0.942	0.985	0.982	0.982	1	0.991	0.958	0.993	0.981	0.911	0.96	0.948	0.976	0.958	0.988	0.974	0.992
**S6**	0.965	0.993	0.987	0.988	0.991	1	0.981	0.993	0.993	0.883	0.938	0.915	0.961	0.945	0.995	0.978	0.993
**S7**	0.989	0.983	0.971	0.97	0.958	0.981	1	0.986	0.899	0.955	0.941	0.973	0.962	0.993	0.981	0.995	0.977
**S8**	0.953	0.993	0.980	0.979	0.993	0.993	0.971	1	0.986	0.899	0.955	0.941	0.973	0.962	0.993	0.981	0.995
**S9**	0.978	0.989	0.986	0.987	0.981	0.993	0.988	0.986	1	0.881	0.934	0.906	0.964	0.942	0.993	0.983	0.993
**S10**	0.849	0.898	0.917	0.91	0.911	0.883	0.833	0.899	0.881	1	0.916	0.922	0.954	0.854	0.906	0.997	0.918
**S11**	0.868	0.932	0.921	0.921	0.96	0.938	0.89	0.955	0.934	0.916	1	0.98	0.969	0.982	0.934	0.939	0.956
**S12**	0.825	0.911	0.906	0.905	0.948	0.915	0.851	0.941	0.906	0.922	0.98	1	0.962	0.968	0.915	0.919	0.936
**S13**	0.931	0.967	0.973	0.971	0.976	0.961	0.932	0.973	0.964	0.954	0.969	0.962	1	0.953	0.971	0.969	0.984
**S14**	0.875	0.935	0.915	0.918	0.958	0.945	0.909	0.962	0.942	0.854	0.982	0.968	0.953	1	0.935	0.955	0.954
**S15**	0.973	0.996	0.993	0.991	0.988	0.995	0.983	0.993	0.993	0.906	0.934	0.915	0.971	0.935	1	0.983	0.996
**S16**	0.957	0.975	0.970	0.969	0.974	0.978	0.970	0.981	0.983	0.997	0.939	0.919	0.969	0.955	0.983	1	0.987
**Reference**	0.969	0.994	0.991	0.990	0.992	0.993	0.977	0.995	0.993	0.918	0.956	0.936	0.984	0.954	0.996	0.987	1

Supplementaty Note 6 : Funding information

Funding 1: Guangdong Provincial Bureau of Traditional Chinese Medicine

The Secondary Development Project of Famous and High-quality Chinese Patent Medicine in Guangdong Province

Contract

Project name: Secondary development of Zishen Yutai pills, a famous and high-quality Chinese patent medicine

Project start and end time: January 2017 to December 2019

Responsible unit: Sun Yat-sen University

Mailing address: 135 Xingang west road, Guangzhou.

Postal code: 510275.

Project leader: Yang Dongzi.

Project contact: Li Yu.

E-mail:

Made by Guangdong Bureau of Traditional Chinese Medicine
September 2016

广东省名优中成药二次开发项目

合　　同　　书

项目名称：名优中成药滋肾育胎丸的二次开发

项目起止时间：2017 年 1 月至 2019 年 12 月

承担单位：中山大学（盖章）

通讯地址：广州市新港西路 135 号

邮政编码：510275

项目负责人：杨冬梓　电话：13922272513

联系人：李予　电话：13660141860

邮箱：yangdz@mail.sysu.edu.cn

广东省中医药局 制

二零一六年九月

Funding 2: Science and Technology Program of Guangzhou Project Contract

Science and Technology Program of Guangzhou Project Contract

Guangzhou Science and Innovation Approval number: (2017) No.107
Project No.: 201704020046

Guangzhou Science and Technology Plan Project Contract

(Funding Classification: One payment prior to project)

Project name: Clinical and experimental study on the application of kidney-tonifying Chinese patent medicine in in vitro fertilization and embryo transfer in advanced maternal age infertile women

Program category: Major innovation project of industry-university-research collaborative

Special issue name: Science and technology research for people's livelihood

Start and end time: 2017-05-01 to 2020-04-30

Responsible Affiliation: Sun Yat-sen Memorial Hospital, Sun Yat-sen University

Organizing Affiliation: Sun Yat-sen University

Responsible department: Division of Social Foundation

Date of Filling in the Form: 2017-01-17 16:48

Made by Guangzhou Science and Technology Innovation Commission
(2016 version)

201704020046

穗科创字〔2017〕107号 批文号：

项目编号：201704020046

广州市科技计划项目
合同书

（前期资助一次性拨付类）

项目名称： 补肾中成药在高龄不孕女性行体外受精与胚胎移植中应用的临床与实验研究

计划类别： 产学研协同创新重大专项

专题名称： 民生科技研究

起止时间： 2017-05-01到2020-04-30

承担单位： 中山大学孙逸仙纪念医院

组织单位： 中山大学

责任处室： 社基处

填表日期： 2017-01-17 16:48

广州市科技计划项目合同书

广州市科技创新委员会制

(2016年版)
