# Supplementary material for: Zishen Yutai Pill increased live births in advanced maternal age women: a randomized clinical trial
Source: Nat Commun. 2025 Dec 24;17:980. doi: 10.1038/s41467-025-67714-4 (PMC12848059; doi:10.1038/s41467-025-67714-4)
Supplement: Supplementary file 2 — Reporting summary [file 41467_2025_67714_MOESM2_ESM.pdf]

nature portfolio

Corresponding author(s): Heping Zhang

Last updated by author(s): Nov 18, 2025

Reporting Summary

Nature Portfolio wishes to improve the reproducibility of the work that we publish. This form provides structure for consistency and transparency in reporting. For further information on Nature Portfolio policies, see our Editorial Policies and the Editorial Policy Checklist.

Statistics

For all statistical analyses, confirm that the following items are present in the figure legend, table legend, main text, or Methods section.

n/a	Confirmed	
☐	☒	The exact sample size (*n*) for each experimental group/condition, given as a discrete number and unit of measurement
☒	☐	A statement on whether measurements were taken from distinct samples or whether the same sample was measured repeatedly
☐	☒	The statistical test(s) used AND whether they are one- or two-sided<br>*Only common tests should be described solely by name; describe more complex techniques in the Methods section.*
☐	☒	A description of all covariates tested
☐	☒	A description of any assumptions or corrections, such as tests of normality and adjustment for multiple comparisons
☐	☒	A full description of the statistical parameters including central tendency (e.g. means) or other basic estimates (e.g. regression coefficient) AND variation (e.g. standard deviation) or associated estimates of uncertainty (e.g. confidence intervals)
☒	☐	For null hypothesis testing, the test statistic (e.g. *F*, *t*, *r*) with confidence intervals, effect sizes, degrees of freedom and *P* value noted<br>*Give P values as exact values whenever suitable.*
☒	☐	For Bayesian analysis, information on the choice of priors and Markov chain Monte Carlo settings
☒	☐	For hierarchical and complex designs, identification of the appropriate level for tests and full reporting of outcomes
☐	☒	Estimates of effect sizes (e.g. Cohen's *d*, Pearson's *r*), indicating how they were calculated

Our web collection on statistics for biologists contains articles on many of the points above.

Software and code

Policy information about availability of computer code

Data collection	De-identified individual data are available at http://github.com/hepingzhangyale/Zishen-Yutai-Pill. Supporting documents are provided in one PDF.
Data analysis	SPSS 19.0. Code is also available at http://github.com/hepingzhangyale/Zishen-Yutai-Pill.

For manuscripts utilizing custom algorithms or software that are central to the research but not yet described in published literature, software must be made available to editors and reviewers. We strongly encourage code deposition in a community repository (e.g. GitHub). See the Nature Portfolio guidelines for submitting code & software for further information.

Data

Policy information about availability of data

All manuscripts must include a data availability statement. This statement should provide the following information, where applicable:

- Accession codes, unique identifiers, or web links for publicly available datasets
- A description of any restrictions on data availability
- For clinical datasets or third party data, please ensure that the statement adheres to our policy

De-identified individual participant data are provided as Source Data file. Additional information and results related to the trial can be found at clinicaltrials.gov under accession: NCT03703700(https://www.clinicaltrials.gov/). Study protocol, statistical analysis plan and informed consent form are available in Supplement Files.

Research involving human participants, their data, or biological material

Policy information about studies with human participants or human data. See also policy information about sex, gender (identity/presentation), and sexual orientation and race, ethnicity and racism.

Reporting on sex and gender	1467 participants were enrolled in the trial, all of whom were female as a sex assigned at birth. In all included patients, sex coincides with gender.
Reporting on race, ethnicity, or other socially relevant groupings	No reporting or analyses based on race, ethnicity or other socially-relevant groupings were performed in this study.
Population characteristics	The following inclusion and exclusion criteria were set forth according to our previous research, with modifications in age and body mass index (BMI).<br>The trial included infertile women planning to undergo IVF/ICSI-ET, aged 35 to 42 years, with a body mass index (BMI) below 28 kg/m2, who had both ovaries present and provided written informed consent.
Recruitment	Recruitment was conducted from March 4, 2019 to October 14, 2023. We screened 1,673 women after pre-screening hospital records. A total of 1,467 women (87.7%) were included in the intention-to-treat analysis, who were randomly assigned to receive ZYP (734 women) or placebo (733 women) .
Ethics oversight	Ethical approval was obtained from the Ethics Committee of Sun Yat-Sen Memorial Hospital, Guangzhou, China (2017 Reproduction Ethnic Approval No.2) and all participating centres. Written informed consent was obtained from all participants.

Note that full information on the approval of the study protocol must also be provided in the manuscript.

Field-specific reporting

Please select the one below that is the best fit for your research. If you are not sure, read the appropriate sections before making your selection.

☒ Life sciences ☐ Behavioural & social sciences ☐ Ecological, evolutionary & environmental sciences

For a reference copy of the document with all sections, see nature.com/documents/nr-reporting-summary-flat.pdf

Life sciences study design

All studies must disclose on these points even when the disclosure is negative.

Sample size	In our previous clinical research, the LBR was 0.42 per embryo transfer in ZYP treated group and 0.33 per embryo transfer in placebo control group among AMA women. By assuming the same LBRs, 454 women per arm could provide 80% power with a significance level of 0.05. According to our previous study, the dropout rate before ET was 15% and the cycle cancellation was 23% in AMA women. While computing the sample size for this trial, we considered a total rate of 38% (dropout and cancellation). Therefore, we calculated that at least 733 participants in each group were required.
Data exclusions	The intention-to-treat analysis was performed including all randomized participants in their originally assigned groups. No data were excluded in ITT analysis.<br>The per-protocol analysis was performed, excluding those who had major protocol deviation(s), canceled cycle or did not complete the pre-set minimum exposure dosage of the assigned study drug (at least 80% compliance), from the ITT population.
Replication	This is a prospectively designed randomized clinical trial. The methods of the trial have been described in detail in the manuscript and study protocol for replication.
Randomization	Participants were randomly assigned in a 1:1 ratio to either the ZYP or placebo arm using the permuted block randomization method. The randomization was stratified by the age (35-37, 38-39 and 40-42) by following the Human Fertilisation and Embryology Authority. Data management was carried out by an independent third-party company (Guangzhou Evidence-Based Medicine Tech Co., Ltd), including handled randomization, electronic case report form (eCRF) establishment and maintenance, drug dispensing management through a web-based Interactive Response Technology (IRT) system.
Blinding	Throughout the trial, participants, clinical investigators, and staff were blinded to treatment allocation. Both ZYP and placebo were manufactured and provided by Guangzhou Baiyunshan Zhongyi Pharmaceutical Co. Ltd. All intervention drugs were identical in packaging, appearance, and odor.<br>In our protocol, unblinding was permitted in the event of a medical emergency. However, throughout the whole trial, blinding remained intact as no instances of such emergencies occurred. The allocation was disclosed by the statistician managing the IRT upon retrieval of all live birth data.

Behavioural & social sciences study design

All studies must disclose on these points even when the disclosure is negative.

Study description

Research sample

Sampling strategy

Data collection

Timing

Data exclusions

Non-participation

Randomization

Ecological, evolutionary & environmental sciences study design

All studies must disclose on these points even when the disclosure is negative.

Study description

Research sample

Sampling strategy

Data collection

Timing and spatial scale

Data exclusions

Reproducibility

Randomization

Blinding

Did the study involve field work? ☐ Yes ☐ No

Field work, collection and transport

Field conditions

Location

Access & import/export

Disturbance

Reporting for specific materials, systems and methods

We require information from authors about some types of materials, experimental systems and methods used in many studies. Here, indicate whether each material, system or method listed is relevant to your study. If you are not sure if a list item applies to your research, read the appropriate section before selecting a response.

Materials & experimental systems

n/a	Involved in the study
☒	☐ Antibodies
☒	☐ Eukaryotic cell lines
☒	☐ Palaeontology and archaeology
☒	☐ Animals and other organisms
☐	☒ Clinical data
☒	☐ Dual use research of concern
☒	☐ Plants

Methods

n/a	Involved in the study
☒	☐ ChIP-seq
☒	☐ Flow cytometry
☒	☐ MRI-based neuroimaging

Antibodies

Antibodies used

Validation

Eukaryotic cell lines

Policy information about cell lines and Sex and Gender in Research

Cell line source(s)

Authentication

Mycoplasma contamination

Commonly misidentified lines
(See ICLAC register)

Palaeontology and Archaeology

Specimen provenance

Specimen deposition

Dating methods

☐ Tick this box to confirm that the raw and calibrated dates are available in the paper or in Supplementary Information.

Ethics oversight

Note that full information on the approval of the study protocol must also be provided in the manuscript.

Animals and other research organisms

Policy information about studies involving animals; ARRIVE guidelines recommended for reporting animal research, and Sex and Gender in Research

Laboratory animals

Wild animals

Reporting on sex

Field-collected samples

Ethics oversight

Note that full information on the approval of the study protocol must also be provided in the manuscript.

Clinical data

Policy information about clinical studies

All manuscripts should comply with the ICMJE guidelines for publication of clinical research and a completed CONSORT checklist must be included with all submissions.

Clinical trial registration	The trial was registered at ClinicalTrials.gov, NCT03703700.
Study protocol	The study protocol has been attached in supplementary information.
Data collection	Participants were enrolled at 12 sites in China. Recruitment was conducted from March 4, 2019 to October 14, 2023. Follow-up was completed in September, 2024.<br>Clinical research coordinator (CRC) was responsible for entering the data of CRF into the database. All data required on the eCRF had to be recorded. Each blank of the eCRF had to be completed, and all items had to be filled in. If the item was "not done", then selected "ND". Double data entry was performed by two independent CRCs separately to make two databases, which were compared by the data manager to generate a list of inconsistent data. Any inconsistencies between the two databases were identified by the data manager, and queried via the eCRF system until final settlement.<br>Data were verified by independent statisticians to generate the data query list according to the data verification plan (DVP). Then clinical research associate (CRA) asked the corresponding investigator to answer the queries in the form, after which the form with answers were given back to the data manager. The database were revised based on these forms.<br>The database managers, Dr. Yu Li, biostatistician Prof. Heping Zhang, and project leader Prof. Dongzi Yang, were in charge of the eCRF records, and were responsible for the assignment of the jurisdiction to the users.<br>Audit were performed during the whole trial process to ensure the data quality. The investigator team compared data in the database against medical record in the hospital information system (HIS) to ensure accuracy.
Outcomes	Following our previous report, live birth rate was the primary outcome. Live birth was defined as delivery of any viable infants after 28 weeks of gestation in a fresh embryo transfer cycle.<br>The secondary outcomes included counts and rates of oocytes/embryos (oocytes retrieved, 2 pro-nuclei zygotes, cleavage zygotes, available embryos, high-quality embryos), pregnancy outcomes (rates of biochemical pregnancy, implantation, clinical pregnancy and miscarriage), incidences of maternal, fetal and neonatal complications, and neonate information (newborn birth weight and length, congenital malformation).<br>High-quality embryo was defined according to the day of embryo transfer, following the Istanbul consensus and Gardner criteria, Day 2: 4 cells, cell fragments <10% and no multi-nucleus; Day 3: 8 cells, cell fragments <10%, no multi-nucleus; Day 5: stage 4 blastocyst, grade A inner cell mass, grade A trophectoderm.<br>Biochemical pregnancy was defined as a serum β-hCG level of >50 IU/L. Implantation rate was defined as the number of gestational sacs per number of embryos transferred. Clinical pregnancy was defined as the presence of intrauterine gestation sac with fetal motion (detectable fetal cardiac activity) under transvaginal ultrasonography. Miscarriage rate was analyzed among patients with positive pregnancy tests and patients with clinical pregnancies. Detailed definitions of maternal, fetal and neonatal complications were listed in Supplementary Note 1.

Dual use research of concern

Policy information about dual use research of concern

Hazards

Could the accidental, deliberate or reckless misuse of agents or technologies generated in the work, or the application of information presented in the manuscript, pose a threat to:

No	Yes	
☐	☐	Public health
☐	☐	National security
☐	☐	Crops and/or livestock
☐	☐	Ecosystems
☐	☐	Any other significant area

Experiments of concern

Does the work involve any of these experiments of concern:

No	Yes	
☐	☐	Demonstrate how to render a vaccine ineffective
☐	☐	Confer resistance to therapeutically useful antibiotics or antiviral agents
☐	☐	Enhance the virulence of a pathogen or render a nonpathogen virulent
☐	☐	Increase transmissibility of a pathogen
☐	☐	Alter the host range of a pathogen
☐	☐	Enable evasion of diagnostic/detection modalities
☐	☐	Enable the weaponization of a biological agent or toxin
☐	☐	Any other potentially harmful combination of experiments and agents

Plants

Seed stocks

Novel plant genotypes

Authentication

ChIP-seq

Data deposition

☐ Confirm that both raw and final processed data have been deposited in a public database such as GEO.

☐ Confirm that you have deposited or provided access to graph files (e.g. BED files) for the called peaks.

Data access links
May remain private before publication.

Files in database submission

Genome browser session
(e.g. UCSC)

Methodology

Replicates

Sequencing depth

Antibodies

Peak calling parameters

Data quality

Software

Flow Cytometry

Plots

Confirm that:

☐ The axis labels state the marker and fluorochrome used (e.g. CD4-FITC).

☐ The axis scales are clearly visible. Include numbers along axes only for bottom left plot of group (a 'group' is an analysis of identical markers).

☐ All plots are contour plots with outliers or pseudocolor plots.

☐ A numerical value for number of cells or percentage (with statistics) is provided.

Methodology

Sample preparation

Instrument

Software

Cell population abundance

Gating strategy

☐ Tick this box to confirm that a figure exemplifying the gating strategy is provided in the Supplementary Information.

Magnetic resonance imaging

Experimental design

Design type

Design specifications

Behavioral performance measures

Acquisition

Imaging type(s)

Field strength

Sequence & imaging parameters

Area of acquisition

Diffusion MRI ☐ Used ☐ Not used

Preprocessing

Preprocessing software

Normalization

Normalization template

Noise and artifact removal

Volume censoring

Statistical modeling & inference

Model type and settings

Effect(s) tested

Specify type of analysis: ☐ Whole brain ☐ ROI-based ☐ Both

Statistic type for inference

(See Eklund et al. 2016)

Correction

Models & analysis

n/a	Involved in the study
☐	☐ Functional and/or effective connectivity
☐	☐ Graph analysis
☐	☐ Multivariate modeling or predictive analysis

Functional and/or effective connectivity

Graph analysis

Multivariate modeling and predictive analysis
